# Self-Tuning Deep Brain Stimulation Controller for Suppression of Beta Oscillations: Analytical Derivation and Numerical Validation

**DOI:** 10.3389/fnins.2020.00639

**Published:** 2020-06-30

**Authors:** John E. Fleming, Jakub Orłowski, Madeleine M. Lowery, Antoine Chaillet

**Affiliations:** ^1^Neuromuscular Systems Laboratory, UCD School of Electrical & Electronic Engineering, University College Dublin, Dublin, Ireland; ^2^Laboratoire des Signaux et Systèmes, Université Paris-Saclay, CNRS, CentraleSupélec, Gif-sur-Yvette, France; ^3^Institut Universitaire de France, Paris, France

**Keywords:** deep brain stimulation, self-tuning, Parkinson's disease, beta-band oscillations, closed-loop, adaptive control

## Abstract

Closed-loop control strategies for deep brain stimulation (DBS) in Parkinson's disease offer the potential to provide more effective control of patient symptoms and fewer side effects than continuous stimulation, while reducing battery consumption. Most of the closed-loop methods proposed and tested to-date rely on controller parameters, such as controller gains, that remain constant over time. While the controller may operate effectively close to the operating point for which it is set, providing benefits when compared to conventional open-loop DBS, it may perform sub-optimally if the operating conditions evolve. Such changes may result from, for example, diurnal variation in symptoms, disease progression or changes in the properties of the electrode-tissue interface. In contrast, an adaptive or “self-tuning” control mechanism has the potential to accommodate slowly varying changes in system properties over a period of days, months, or years. Such an adaptive mechanism would automatically adjust the controller parameters to maintain the desired performance while limiting side effects, despite changes in the system operating point. In this paper, two neural modeling approaches are utilized to derive and test an adaptive control scheme for closed-loop DBS, whereby the gain of a feedback controller is continuously adjusted to sustain suppression of pathological beta-band oscillatory activity at a desired target level. First, the controller is derived based on a simplified firing-rate model of the reciprocally connected subthalamic nucleus (STN) and globus pallidus (GPe). Its efficacy is shown both when pathological oscillations are generated endogenously within the STN-GPe network and when they arise in response to exogenous cortical STN inputs. To account for more realistic biological features, the control scheme is then tested in a physiologically detailed model of the cortical basal ganglia network, comprised of individual conductance-based spiking neurons, and simulates the coupled DBS electric field and STN local field potential. Compared to proportional feedback methods without gain adaptation, the proposed adaptive controller was able to suppress beta-band oscillations with less power consumption, even as the properties of the controlled system evolve over time due to alterations in the target for beta suppression, beta fluctuations and variations in the electrode impedance.

## 1. Introduction

Deep brain stimulation (DBS) is a clinically effective treatment used in patients with advanced Parkinson's disease (PD) to supplement or replace pharmacological treatment of symptoms. It consists of high-frequency stimulation of neurons within the basal ganglia, with the subthalamic nucleus and globus pallidus being the most common targets, using a chronically implanted electrode and a subcutaneous pulse generator. At present, DBS is delivered clinically in an open-loop fashion where stimulation parameters remain fixed over time. This approach, however, may lead to overstimulation, inducing side effects and shortening battery life. Regular retuning of parameters is also required, involving a time-consuming trial and error process (Lozano et al., 2019).

The search for new approaches to improve the attenuation of patient symptoms and minimize side effects while increasing battery life has motivated a growing interest in closed-loop DBS over the last several years (Santaniello et al., 2010; Rosin et al., 2011; Santos et al., 2011; Carron et al., 2013; Beuter et al., 2014; Shah et al., 2018; Eitan et al., 2019). In a closed-loop paradigm, the stimulation waveform is modified based on specific biomarkers which are used as a surrogate measure of symptom severity. To date, beta-band (13–30 Hz) activity in the STN local field potential (LFP) has been one of the most widely investigated biomarkers for closed-loop DBS during Parkinson's disease (Parastarfeizabadi and Kouzani, 2017). Increased beta-band power in the STN LFP is correlated with motor impairment symptoms in Parkinson's disease, and its suppression, due to medication or high frequency DBS, with improved motor performance (Kühn et al., 2006, 2008; Hammond et al., 2007; Eusebio et al., 2011).

Closed-loop stimulation utilizing LFP beta-band power has been experimentally validated in patients for short periods (Rosin et al., 2011; Little et al., 2013, 2016; Rosa et al., 2015; Arlotti et al., 2018; Velisar et al., 2019), however, longer term investigations have yet to be conducted. The improvement in symptoms has been comparable to that with conventional open-loop DBS while the energy required has been substantially reduced. In a number of studies, the stimulation signal has been delivered in an “on-off” fashion, switching the stimulation on or off depending on whether or not the biomarker exceeded a specified threshold (Little et al., 2013, 2016). Using this approach, suitable stimulation parameters must be first identified, as in the case of open-loop DBS. Using two thresholds, Velisar et al. (2019) increased or decreased stimulation amplitude to maintain the biomarker within a given range. Proportional feedback approaches, where the stimulation amplitude is proportional to the measured biomarker, have also shown potential in both clinical (Rosa et al., 2015; Arlotti et al., 2018) and computational studies (Tukhlina et al., 2007; Chaillet et al., 2017; Popovych and Tass, 2019). Clinical studies in patients have confirmed that proportional stimulation responding to slowly changing beta-band LFP activity is not only effective and well-tolerated by patients, but might also help avoid stimulation-induced dyskinesia when patients are on medication (Rosa et al., 2015; Arlotti et al., 2018).

Similar to on-off strategies, which utilize fixed stimulation parameters, proportional feedback requires a fixed controller gain parameter to be identified where the stimulation amplitude at a given time is controlled by this gain and an estimated biomarker value. Identification of the controller gain parameter is a potentially complicated and time-consuming postoperative process. Furthermore, although the selected gain may be suitable for the operating point at which it was initially set, it may provide suboptimal performance and require retuning when properties of the system change, for example in response to disease progression or changes in the properties of the electrode-tissue interface.

To address this problem, we propose a self-tuning control strategy inspired by adaptive control theory, where the value of the controller gain evolves based on the measured pathological activity. The controller gain automatically increases until the detected pathological oscillations are sufficiently suppressed and then begins to dissipate when the oscillation amplitude is low enough. In this manner, the proportional controller self-adapts its gain to the lowest value that guarantees suppression of the oscillations to the desired level. Using tools from control theory, namely Lyapunov-Krasovskii analysis, we mathematically show in a firing-rate model that the proposed controller guarantees disruption of the pathological oscillations provided that the internal coupling within the GPe is sufficiently weak. The controller is derived first under the assumption of endogenously generated oscillations, arising from increased coupling within the STN-GPe network, and is then extended to the case where oscillatory activity arises in the STN-GPe loop due to exogenous cortical inputs.

While firing-rate models provide a means to represent the activity of the network in a mathematically tractable manner, they lack the physiological detail that enables them to be easily related to the underlying processes at the cellular level. To overcome this limitation, we computationally test its performance in a more physiologically relevant context, by implementing it in a network of conductance-based neuron models. We first demonstrate through simulations that the firing-rate and conductance-based network models studied here have similar characteristics in terms of the emergence of oscillations as a function of connection strength between STN and GPe, and that both models have similar qualitative frequency responses. Firing-rate model parameters are then identified based on data obtained from simulations of the conductance-based model to link the two models and demonstrate that the latter fulfills the theoretical criterion of low internal GPe connectivity. Finally, the ability of the adaptive controller to “self-tune” to maintain the suppression of pathological oscillations is assessed numerically in three different scenarios: changing the target suppression level, modifying the background beta activity in the network, and varying the electrode impedance.

## 2. Materials and Methods

### 2.1. Firing-Rate Model

The firing-rate model, which we derive the self-tuning DBS strategy and mathematically prove its efficacy, is inspired by the STN-GPe loop model originally proposed in Nevado-Holgado et al. (2010) to study the emergence of pathological beta oscillations observed in the parkinsonian basal ganglia. The model is defined as follows:

(1a) $$\begin{array}{rc} \tau_{1}{ẋ}_{1}\left(t\right) & =-x_{1}\left(t\right)+S_{1}(c_{11}x_{1}\left(t-\delta_{11}\right)-c_{12}x_{2}\left(t-\delta_{12}\right)+b_{1}u_{1}\left(t\right)) \end{array}$$

(1b) $$\begin{array}{rc} \tau_{2}{ẋ}_{2}\left(t\right) & =-x_{2}\left(t\right)+S_{2}(c_{21}x_{1}\left(t-\delta_{21}\right)-c_{22}x_{2}\left(t-\delta_{22}\right)-b_{2}u_{2}\left(t\right)). \end{array}$$

The instantaneous firing activities (in pulses per second) of the STN and GPe, are respectively represented by *x*_1_(*t*) and *x*_2_(*t*). τ_1_, τ_2_ > 0 are time constants. For each *i, j* ∈ {1, 2}, the constant *c*_*ij*_ ≥ 0 represents the synaptic connection strength from population *j* to population *i* and δ_*ij*_ ≥ 0 is a time delay that occurs due to finite velocity of axonal and synaptic transmission from population *j* to population *i*. *u*_1_ and *u*_2_ represent the influence of cortical (into STN) and striatal (into GPe) inputs to the system, respectively, modulated by the synaptic weights *b*_1_ ≥ 0 and *b*_2_ ≥ 0. All the coupling constants *c*_*ij*_ and *b*_*j*_ being non-negative, the sign represents whether the neurons in the presynaptic population have excitatory (STN and cortex) or inhibitory (GPe and striatum) effect on the postsynaptic one. The activation functions *S*_1_ and *S*_2_ encode the response of the neuronal populations to their input. Although they were considered in Nevado-Holgado et al. (2010) as sigmoids, the theoretical analysis provided below allows them to be any increasing function with bounded derivative.

#### 2.1.1. Self-Tuning Controller Derivation

It was shown in Nevado-Holgado et al. (2010) and Pasillas-Lépine (2013) that if the coupling weights *c*_12_ and *c*_21_ between STN and GPe are sufficiently high, the model exhibits sustained endogenous oscillations, which fall in the beta frequency band for appropriate values of the model parameters. The controller is first derived and assessed under that assumption and is then further explored in the case where oscillations within the loop arise from exogenous inputs to the STN-GPe network.

Under the assumption that the cortical and striatal inputs are constant ($u\left(t\right)=(u_{1}(t),u_{2}\left(t\right){)}^{T}=ū$), consider the change of variables *u* ↤ *u* − ū, and *x* ↤ *x* − $\overset{-}{x}$, where $\bar{x}$ is an equilibrium value of $x={\left(x_{1},x_{2}\right)}^{T}$ for the input ū, whose existence is guaranteed by Pasillas-Lépine (2013). Adding a feedback μ(*t*) representing the influence of artificial stimulation (DBS) on STN and shifting the activation functions *S*_*j*_ such that *S*_*j*_(0) = 0 for *j* ∈ {1, 2}, we obtain the following dynamics:

(2a) $$\begin{array}{rc} \tau_{1}{ẋ}_{1}\left(t\right) & =-x_{1}\left(t\right)+S_{1}(c_{11}x_{1}\left(t-\delta_{11}\right)-c_{12}x_{2}\left(t-\delta_{12}\right)+\mu \left(t\right)) \end{array}$$

(2b) $$\begin{array}{rc} \tau_{2}{ẋ}_{2}\left(t\right) & =-x_{2}\left(t\right)+S_{2}(c_{21}x_{1}\left(t-\delta_{21}\right)-c_{22}x_{2}\left(t-\delta_{22}\right)). \end{array}$$

The model described by (2) has been studied by Pasillas-Lépine et al. (2013) and Haidar et al. (2016), who showed that a proportional feedback acting only on STN is capable of disrupting exaggerated oscillations and stabilizing the system. More precisely, assuming that the inner GPe interconnections are sufficiently weak, namely:

(3) $$\begin{array}{r} c_{22}\ell_{2}<1, \end{array}$$

where ℓ_2_ denotes the maximum slope of the GPe activation function *S*_2_, it was demonstrated in those papers that the system (2) is asymptotically stable under proportional feedback from the STN to itself, namely:

(4) $$\begin{array}{r} \mu \left(t\right)=-\theta x_{1}\left(t\right), \end{array}$$

where the proportional gain θ > 0 should be chosen to be sufficiently large.

The original result was obtained using linearization techniques. Since then, it has been extended to take full account of the nonlinear effects induced by the activation functions *S*_*j*_ (Chaillet et al., 2017, 2019). In particular, it was shown in Chaillet et al. (2019), using Lyapunov-Krasovskii methodology, that the network described in (2) in closed loop with (4) is globally exponentially stable, provided that θ is above some minimum value θ^*^ > 0 and condition (3) is satisfied. Global exponential stability of the origin means that there exist η, γ > 0 such that the solutions of (2) satisfy

(5) $$\begin{array}{r} |x\left(t\right)|\leq \eta ||x_{0}||e^{-\gamma t},\;\forall t\geq 0, \end{array}$$

for any initial state *x*_0_. The model being a delay differential equation, the state here is not a point in ℝ^2^, but rather a history function defined as *x*_*t*_(*s*) = *x*(*t* + *s*) for all *s* ∈ [−δ, 0], where δ denotes the maximum delay δ_*ij*_ involved in the dynamics. This state (hence, the initial state) belongs to the set of all continuous functions from [−δ, 0] to ℝ^2^ and we employ the norm $\left||x_{t}||=\underset{s\in \left[-\delta ,0\right]}{\max}|x\left(t+s\right)\right|$ on this functional set. Global exponential stability is thus a very strong stability property, as it imposes an exponential convergence to the equilibrium and a transient overshoot proportional to the magnitude of the initial state, no matter where the system initially lies. Imposing such a property on the firing-rate model (2) impedes the existence of steady-state pathological oscillations. Global exponential stability is also known to induce robustness properties with respect to exogenous inputs for a wide class of systems (Yeganefar et al., 2008), which may prove useful for the problem considered here due to the inherent imprecision and variability of biological models.

One of the major limitations of the stimulation strategy proposed above is that we do not know a priori the value of the minimum effective gain θ^*^. In Chaillet et al. (2019), the following estimate of θ^*^ was proposed:

$$\begin{array}{l} \theta^{*}\leq 8\left(c_{11}^{2}+\frac{4c_{21}^{2}c_{12}^{2}}{{\left(1-c_{22}\right)}^{2}}\right), \end{array}$$

but it is a conservative approximation. Moreover, this value depends on the connection parameters *c*_*ij*_ that would be very hard to estimate accurately in practical applications, due to the high level of abstraction of the considered model.

The alternative to pure proportional control that we propose here is inspired from adaptive control theory and involves updating the gain parameter θ based on the measured state of the system, namely:

(6a) $$\begin{array}{rc} \mu \left(t\right) & =-\theta \left(t\right)x_{1}\left(t\right) \end{array}$$

(6b) $$\begin{array}{rc} \tau_{\theta }\overset{∙}{\theta }\left(t\right) & =|x_{1}\left(t\right)|-\sigma \theta \left(t\right), \end{array}$$

where σ, τ_θ_ > 0 are control parameters governing how fast the control gain reacts to changes in the system.

First note that, similar to (4), this control law requires recording of the STN activity only. In the same way, it involves only stimulation of the STN. In practice, these constitute important features in terms of limited insertion of measurement and stimulation electrodes.

The idea behind the adaptive law (6) is simple. As long as *x*_1_ is not at zero (meaning that STN activity has not reached its equilibrium), the term |*x*_1_(*t*)| ≥ 0 increases the proportional gain θ. With that, θ(*t*) will eventually overpass θ^*^ (no matter what its precise value is) and cause the state to converge to zero exponentially. On the other hand, the dissipation (or leakage) term −σθ(*t*) decreases the value of θ whenever σθ(*t*) ≥ |*x*_1_(*t*)|, due to either a too high value reached by the gain θ or because *x*_1_ has reached a sufficiently low value (as desired). These balanced effects are designed in such a way that the control law automatically adjusts its gain around the (unknown) value θ^*^.

The dissipation term −σθ(*t*) present in (6b) is in the spirit of what is known in the control theory literature as the “σ-modification” (Ioannou and Kokotovic, 1984). It was introduced to increase robustness of adaptive control to external disturbances and unmodeled dynamics. It has been shown to guarantee that all the closed-loop signals are bounded and that their mean values converge to a residual set, whose size can be made arbitrarily small with an appropriate choice of σ (Ioannou and Fidan, 2006), even for certain classes of nonlinear systems (Fradkov et al., 1999). Until recently, this methodology was confined to delay-free systems but, for the purpose of the present study, we have extended it to nonlinear time-delay systems (Orłowski, 2019). More precisely, we have the following result (the interested reader is referred to Orłowski, 2019 for more details on the mathematical aspects of this result).

**Proposition**. *Let* ℓ_*i*_ *denote the maximum slope of the activation functions* *S*_*i*_ *and let* ${\tilde{\theta }}_{0}=\theta_{0}-\theta^{*}$. Under the condition that ℓ_2_*c*_22_ < 1*, there exists* *q* > 0 *such that, for any* σ ≥ 0 *small enough and any initial conditions* *x*_0_ *and* θ_0_*, the solution of system (2) in closed loop with (6) is bounded and satisfies the following property for all* *t, T* ≥ 0*:*

$$\frac{1}{T}\overset{t+T}{\underset{t}{\int }}|x\left(\tau \right)|\text{d}\tau \leq \frac{q}{T}\left(||x_{0}||+\min\left\{{\tilde{\theta }}_{0};0\right\}{\tilde{\theta }}_{0}+1\right)+q\sigma .$$

This statement ensures that solutions are bounded and the system is “stable in the mean.” This latter property guarantees that the mean value of the solution, taken over a sufficiently long time window *T*, converges to a neighborhood proportional to σ, regardless of the initial state. Since σ is a tunable parameter in our controller, this means we can arbitrarily decrease the average amplitude of steady-state oscillations by picking a sufficiently small σ (picking σ as zero would annihilate steady-state oscillations, but would impede the ability to decrease the proportional gain θ whenever possible). The key assumption under which this stabilization is made possible is that ℓ_2_*c*_22_ < 1, meaning that the GPe self-coupling should be reasonably low. More discussion on this assumption and its biological meaning is provided in section 4.2.

In order to selectively attenuate pathological oscillations, with moderate effect on other frequency bands, we propose the following frequency-sensitive version of (6):

(7a) $$\begin{array}{rc} \mu \left(t\right) & =-\theta \left(t\right)x_{1}\left(t\right) \end{array}$$

(7b) $$\begin{array}{rc} \tau_{\theta }\overset{∙}{\theta }\left(t\right) & =\beta \left(x_{1t}\right)-\sigma \theta \left(t\right), \end{array}$$

where β(*x*_1*t*_) ≥ 0 is a biomarker detection function. β is implemented as the peak-to-peak amplitude of a bandpass-filtered signal (Butterworth filter, order 5, 15–30 Hz) from *t* − 500 ms to *t*.

This version is similar in spirit to the self-tuning controller (6), but the increase of the gain θ depends only on the STN activity within the targeted frequency band (beta). In a situation when no beta activity is present in the STN, the leakage term −σθ(*t*) would cause the gain θ(*t*) to converge to zero, in which case no DBS signal would be delivered.

#### 2.1.2. Endogenous and Exogenous Generation of Beta Oscillations

The mathematical derivations in section 2.1.1 assume that external inputs into the STN-GPe loop are constant and pathological oscillations arise in an endogenous manner due to too strong synaptic coupling between STN and GPe (Plenz and Kital, 1999; Nevado-Holgado et al., 2010; Pavlides et al., 2012). However, the origin of beta oscillations is still a subject of much debate and there is increasing evidence supporting a role for an exogenous mechanism in which oscillations originating in the cortex or striatum are transmitted to the STN-GPe network and amplified within the network (Magill et al., 2001; Sharott et al., 2005; Mallet et al., 2008; Tachibana et al., 2011; Corbit et al., 2016).

To simulate oscillations arising due to exogenous inputs to the STN, the synaptic coupling between STN and GPe were taken as sufficiently low such that the firing-rate model is not only stable but also incrementally stable (Chaillet et al., 2013) which implies that its steady-state solutions in response to any *T*-periodic inputs are themselves *T*-periodic. Thus, any oscillatory input (from cortex or striatum) can entrain the STN-GPe network and cause it to oscillate at the same frequency. This observation enables us to identify the frequency characteristics of the STN-GPe network (despite its nonlinear nature) and which frequency bands, if any, are preferably amplified (see section 3.1.2).

In the firing-rate model, the exogenous and endogenous hypotheses of beta oscillations generation thus correspond to two distinct dynamical behaviors: instability for the former and incremental stability (entrainment) for the latter. Both mechanisms are studied in the simulations presented in this paper (**Figures 2**–**5**), using the parameter values presented in Table 1. Additionally, the time constants of the populations were set to τ_1_ = 6 ms, τ_2_ = 14 ms, and the delays in the system were set to δ_12_ = δ_21_ = 6 ms and δ_22_ = 4 ms. The functions *S*_*i*_ were implemented as sigmoids with slope 1

(8) $$\begin{array}{r} S_{i}\left(x\right)=\frac{M_{i}B_{i}}{B_{i}+\exp\left(-4x/M_{i}\right)\left(M_{i}-B_{i}\right)}, \end{array}$$

where the constants were set to *M*_1_ = 300, *M*_2_ = 400, *B*_1_ = 17, *B*_2_ = 75.

**Table 1:** Parameters of the firing-rate model (1) used in numerical simulations to obtain **Figures 2A**, **3A**, **4**, **5**.

	***c*_12_**	***c*_21_**	***c*_22_**	***b*_1_**	***b*_2_**
**Figure 2A** (endogenous)	0–4	0–32	4	8	139.4
**Figure 3A** (exogenous)	1.12	19	0.9	2.42	15.1
**Figure 4** (endogenous)	3	10	0.9	5	139.4
**Figure 5** (exogenous)	1.12	19	0.9	2.42	15.1

### 2.2. Conductance-Based Model

#### 2.2.1. Model and Controller Implementation

**Figure 1:**
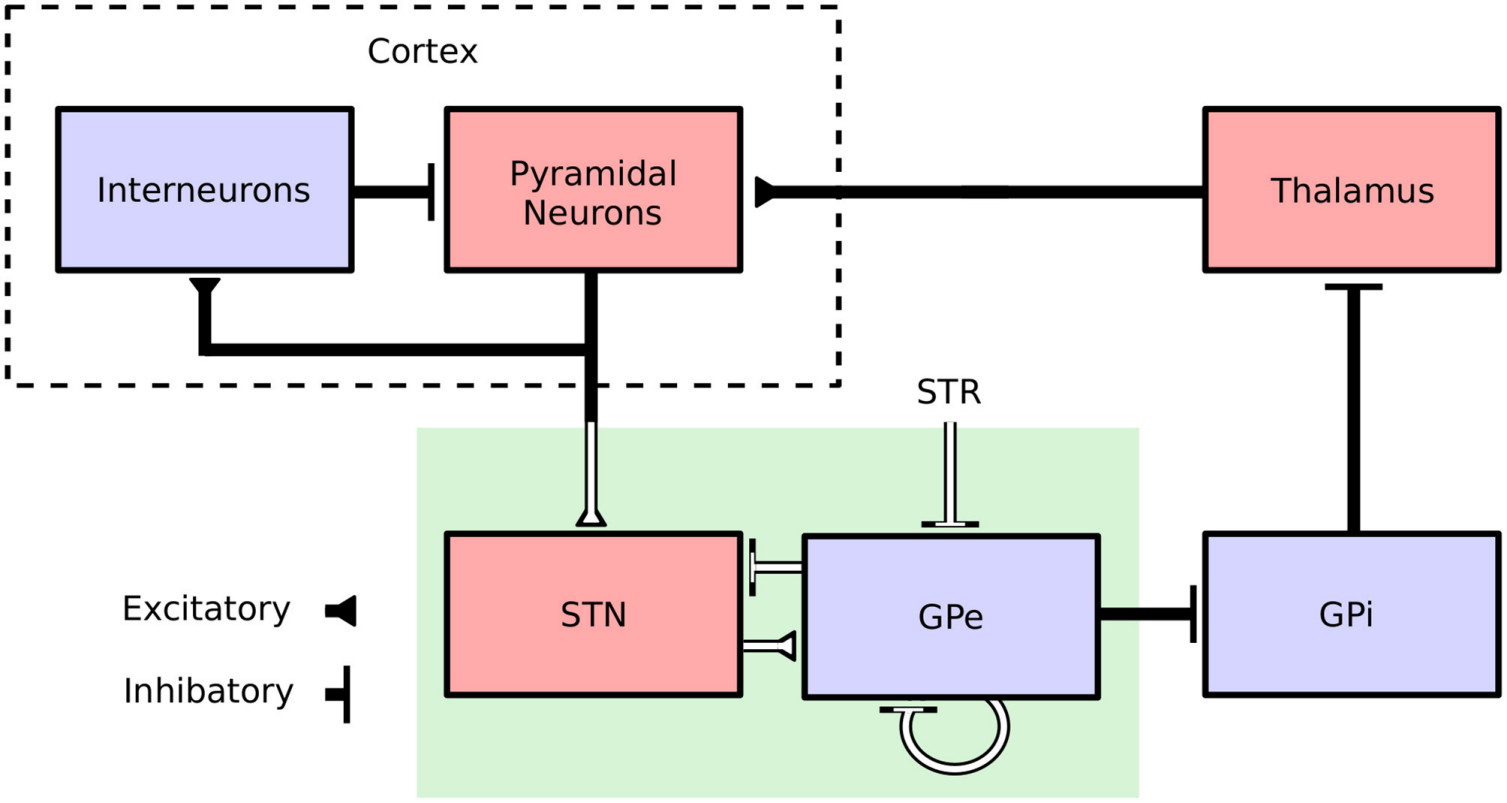
Cortical Basal ganglia network structure. Excitatory and inhibitory populations in the network model are represented in red and blue, respectively. Synaptic connections in the population model are represented by solid black lines. The firing-rate model, representing only a subnetwork of the population model, is highlighted by a green box. White lines correspond to connections which are common to both the population and the firing-rate model.

In view of its self-tuning capacities, induced robustness and limited requirements in terms of recording and stimulation electrodes, the proposed adaptive closed-loop DBS strategy is promising. Nevertheless, the strong abstraction of the model considered, which summarizes the activity of an entire neuronal population by a unique variable (its firing rate), makes it difficult to assess whether this strategy would be effective clinically. To address this, the performance of the proposed controller was assessed in a biophysically realistic network model of the cortical basal ganglia network comprised of conductance-based neurons. The model is presented in Fleming et al. (2020) and consists of a population of multicompartment cortical pyramidal neurons and single compartment models of cortical interneurons as well as STN, GPe, GPi, and thalamic neurons which have been previously validated and used in other modeling studies (Terman et al., 2002; Otsuka et al., 2004; Rubin and Terman, 2004; Pospischil et al., 2008; Hahn and McIntyre, 2010; Foust et al., 2011; Kang and Lowery, 2013, 2014; Kumaravelu et al., 2016). Each population was comprised of 100 neurons, where synaptic connections between neurons were modeled by spike detectors in presynaptic neurons coupled to synapses in postsynaptic neurons by a time delay. Synaptic connections in the model were either excitatory (AMPAergic) or inhibitory (GABAergic) depending on the synapse type (Destexhe et al., 1994). Striatal input to the network was represented as Poisson-distributed spike trains to GPe neurons with a mean firing rate of 3 Hz. An overview of the model structure is presented in Figure 1.

The model captures key features of the cortical basal ganglia network required for simulating clinical implementations of closed-loop DBS including: (i) the extracellular DBS electric field, which is required to accurately model changes in the DBS amplitude, (ii) antidromic and orthodromic activation of STN afferent fibers, and (iii) the STN LFP detected at non-stimulating contacts of the DBS electrode. In Fleming et al. (2020), the model parameters were tuned to match key features observed during experimental investigations of DBS including cortical desynchronization (Li et al., 2012), GPe entrainment (McConnell et al., 2012), and a gradual suppression of beta-band power detected in the STN LFP for increasing stimulation amplitude (Davidson et al., 2016). The model is described in full detail in its original publication (Fleming et al., 2020) and available to download from ModelDB (https://senselab.med.yale.edu/modeldb/) at ascension number 262046.

The oscillatory properties of the model's STN-GPe network were first examined to explore the duality between its oscillatory behavior and that of the firing-rate model. Beta-band activity was then configured to remain fixed, or was varied according to the three numerical scenarios detailed below which may require controller adaptation *in vivo*.

In line with (7), the proposed self-tuning controller was implemented in the conductance-based model to adapt the amplitude of the stimulation waveform as follows:

(9a) $$\begin{array}{rc} \mu \left(t\right) & =\theta \left(t\right)x_{1}\left(t\right) \end{array}$$

(9b) $$\begin{array}{rc} \tau_{\theta }\overset{∙}{\theta }\left(t\right) & =|e\left(t\right)|-\sigma \theta \left(t\right), \end{array}$$

where μ(*t*) is the controller output and represents the instantaneous stimulation amplitude, θ(*t*) is the controller gain, *x*_1_(*t*) is the biomarker measurement [i.e., the average rectified value (ARV) of a 100 ms epoch from the beta-band filtered STN LFP, which was filtered using a fourth order Chebyshev band-pass filter with an 8 Hz bandwidth, centered on 25 Hz as described in Fleming et al., 2020], |*e*(*t*)| is the half-wave rectified error signal calculated as the difference between the measured biomarker and the desired target suppression level, and τ_θ_ and σ represent tuning parameters which were fixed at 100 ms and 0.00875, respectively, for all controller simulations in the conductance-based model.

A key difference with (7) is that the DBS in (9) is delivered as a positive feedback on the biomarker [as indicated by the positive sign in μ(*t*)]. This difference is due to the implementation of DBS in the conductance-based model, whereby increasing DBS amplitude results in a stronger suppression of pathological oscillations.

The model was simulated in the NEURON simulation environment (Hines and Carnevale, 1997) and implemented in Python using the PyNN API package (Davison et al., 2009). The model was numerically integrated using the Crank-Nicholson method with a 0.01 ms timestep for all simulations. Simulations were run on the UCD Sonic high-performance computing cluster.

#### 2.2.2. Numerical Scenarios

The self-tuning controller (9) was tested in three independent scenarios to simulate practical situations in which adaptation of controller parameters *in vivo* may be required to maintain the biomarker (the ARV of LFP beta activity) at a target value. All scenarios were simulated for a 130 s duration.

In the first scenario, background beta-band activity in the model was set to its maximum value for the duration of the simulation while the target level for beta suppression was varied. The beta-band activity (prior to DBS activation) was set by fixing the firing rates of cortical neurons to 26 pulses per second, which resulted in a peak in the LFP power spectrum at 26 Hz. Target values of 10, 0.2, 0.05, 0.15, and 10 μV were then considered over the time intervals 0–10, 10–40, 40–70, 70–100, and 100–130 s, respectively.

In the second scenario, the target value for the LFP biomarker was fixed at 0.1 μV for the duration of the simulation while background beta-band activity in the model was modulated to be low during the 0–10, 40–70, and 100–130 s time intervals and high during the 10–40 and 70–100 s time intervals. The intracellular cortical neuron bias current was varied to shift the mean cortical neuron firing rate between 14 and 26 pulses per second during the low and high beta-band activity periods, respectively. Thus, beta-band activity in the model was modulated to display a 14 Hz peak in the LFP power spectrum during the low beta-band activity periods, which shifted to 26 Hz during the high beta-band activity periods. As the bandwidth of the biomarker filter was centered at 25 Hz, modulation of the background beta-band activity in this manner led to lower beta ARV measurements during the low beta-band activity periods.

The third scenario considered a linear variation of the electrode impedance over the simulation period, while background beta-band activity in the model was fixed at its maximum value and the beta ARV target value remained constant at 0.1 μV. In the simulation, the electrode impedance remained constant at 0.5 kΩ up to *t* = 30 s, after which it was linearly increased to a maximum value of 2.5 kΩ at *t* = 130 s.

#### 2.2.3. Performance Measures

Controller performance was quantified using two measures: the error while tracking the target value and the mean power consumption. The mean squared error (MSE) was utilized to measure the controller's ability to track the target level. It is defined as

(10) $$\begin{array}{r} \text{MSE}=\frac{1}{T_{sim}}\overset{T_{\text{sim}}}{\underset{0}{\int }}e{\left(t\right)}^{2}dt, \end{array}$$

where *T*_sim_ is the simulation duration (*T*_sim_ = 130 s) and *e*(*t*) is the normalized error signal between the measured LFP beta ARV and the target value, as used in (9). For simplicity, the MSE value for the controllers in each scenario are reported as a percentage of the MSE value that was measured in each respective scenario when DBS was off. Power consumption (PC) was measured as

(11) $$\begin{array}{r} \text{PC}=\frac{1}{T_{\text{sim}}}\overset{T_{\text{sim}}}{\underset{0}{\int }}Z_{E}\left(t\right)I_{\text{DBS}}{\left(t\right)}^{2}dt, \end{array}$$

where *Z*_*E*_ is the electrode impedance, assumed to be 0.5 kΩ in the non-varying electrode impedance scenarios, and *I*_DBS_ is the delivered DBS current.

### 2.3. Parameter Identification

One of the necessary conditions for practical stabilization using the adaptive controller, as recalled in section 2.1.1, is that the internal connections within GPe are weak, as expressed by *c*_22_ℓ_2_ < 1 in the firing-rate model. It was, therefore, first checked whether this condition was fulfilled in the conductance-based model.

The value of *c*_22_ from the firing-rate model is related to the maximum conductance of the GPe-GPe synapses ḡ_GPeGPe_, in the conductance-based model, which was set to 0.015 μS. ℓ_2_ is the maximum slope of the GPe activation function *S*_2_. Thus, in order to verify the stabilizability criterion, we need to identify the slope of the GPe activation function in the conductance-based model, as well as the value of *c*_22_ corresponding to the value of ḡ_GPeGPe_ used in the conductance-based model.

To that aim, the GPe neurons of the conductance-based model were disconnected from the rest of the network, the only remaining connections being the excitatory projections from STN to GPe. In the firing-rate model, this translates in the following dynamics:

(12) $$\begin{array}{r} \tau_{2}{ẋ}_{2}\left(t\right)=-x_{2}\left(t\right)+S_{2}(u\left(t\right)+c_{22}x_{2}\left(t-\delta_{22}\right)), \end{array}$$

where *u*(*t*) represents synaptic inputs to GPe from STN. Setting ḡ_GPeGPe_ = 0 in the conductance-based model leads to *c*_22_ = 0 in the firing-rate model, thus yielding

(13) $$\begin{array}{r} \tau_{2}{ẋ}_{2}\left(t\right)=-x_{2}\left(t\right)+S_{2}\left(u\left(t\right)\right). \end{array}$$

We conducted a series of simulations for different values of cortical input to STN and we estimated the firing rate *u*(*t*) of STN and the firing rate *x*_2_(*t*) of GPe. We have rescaled the data to obtain a slope 1, by fitting a linear function and dividing the values by the obtained slope *a* = 1.29. We then fitted a sigmoid of the form (8) to the obtained data and rescaled it by the same factor *a*, to obtain an estimate of the activation function *S*_2_.

Next, we obtained a similar set of data for ḡ_GPeGPe_ = 0.015μS. Using the activation function *S*_2_ determined in the previous step, we found the equilibrium of (12) for different values of *c*_22_ and constant STN input *u* using a numerical solver. We compared the curves obtained for the different values of *c*_22_ with the steady-state data from the conductance-based model, to identify the value that minimized the normalized square error:

(14) $$\begin{array}{r} nLSQ\left(c_{22};\text{STN},\text{GPe}\right)=\frac{\underset{i}{\sum }{\left(f_{c_{22}}\left(\text{STN}\left[i\right]\right)-\text{GPe}\left[i\right]\right)}^{2}}{\underset{i}{\sum }\text{GPe}{\left[i\right]}^{2}}, \end{array}$$

where STN[*i*] and GPe[*i*] represent the firing rate of STN and GPe taken from simulation *i*, and *f*_*c*_22__(STN[*i*]) is the solution of

$$\begin{array}{l} x=S_{2}\left(u+c_{22}x\right) \end{array}$$

for a given *c*_22_ with *u* = STN[*i*], meaning the steady-state solution of (12) for these generated inputs from STN. The best fit was reached for *c*_22_ = 0.35. Since the GPe activation *S*_2_ identified in (8) has maximum slope ℓ_2_ = 1.29, the stabilizability criterion (3) is satisfied.

## 3. Results

### 3.1. Qualitative Comparison Between the Two Models

#### 3.1.1. Endogenous Oscillations

**Figure 2:**
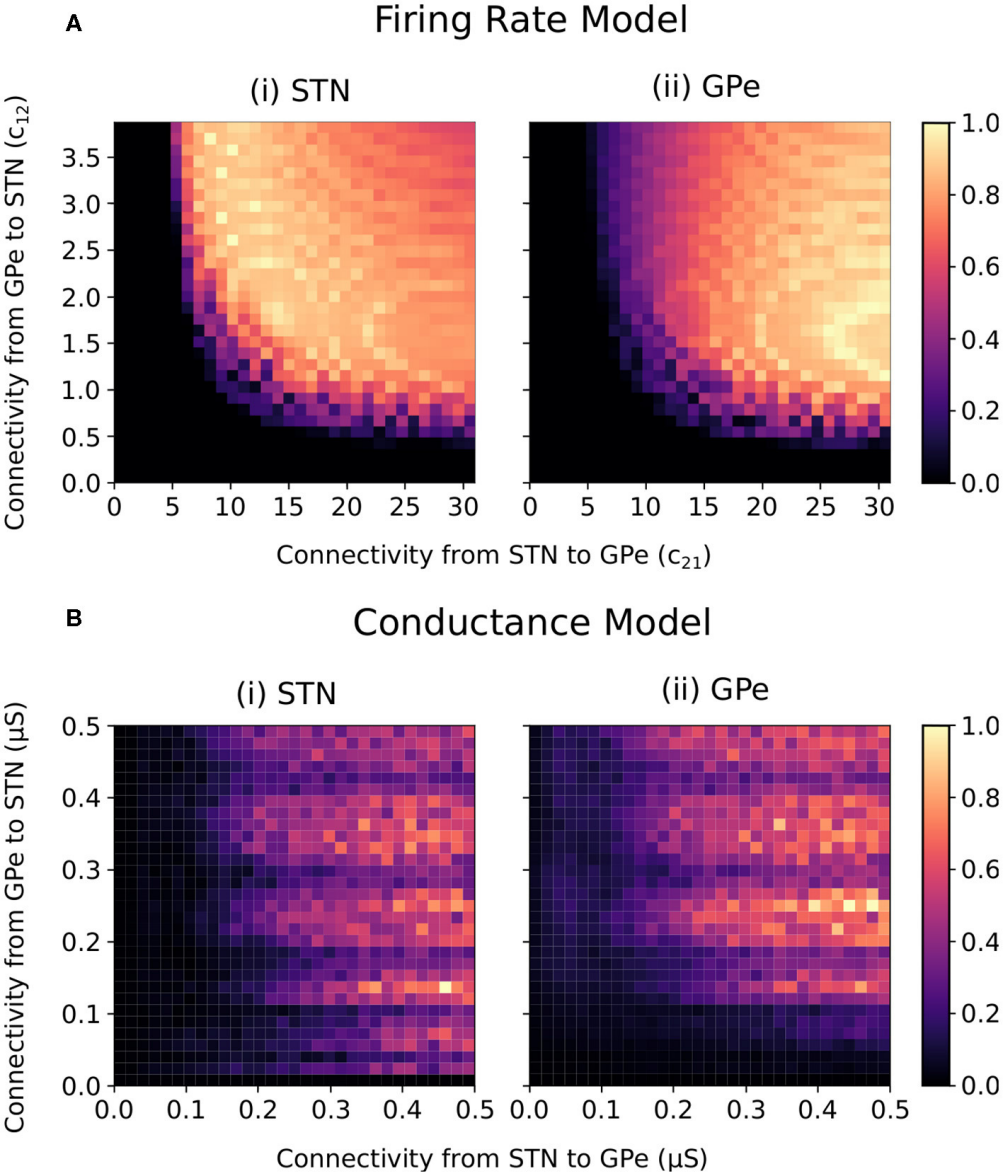
Normalized endogenous beta-band activity in the firing rate and conductance-based models for varying STN and GPe coupling strengths. **(A)** Beta-band oscillation amplitude in the (i) STN and (ii) GPe of the firing rate model. The beta-band oscillation amplitude in each population was estimated for each combination of connectivity parameters (*c*_12_ and *c*_21_) by band-pass filtering the firing rate signals using a fifth order Butterworth filter with 8 Hz bandwidth centered on 20 Hz. The oscillation amplitude in each population was normalized between 0 and 1 in each panel separately. **(B)** Beta-band activity of the (i) STN and (ii) GPe populations in the conductance-based model. Beta-band activity was quantified in each population by integrating the power spectra of the cumulative spike trains for each population between 16 and 24 Hz. The power for each population was normalized between 0 and 1 in each panel separately.

For constant striatal and cortical inputs, beta-band oscillations emerged in the firing-rate model when the STN-GPe and GPe-STN connectivity strength was sufficiently increased (Figure 2A). In the conductance-based model, the intensity of beta power in the spectra of the cumulative STN and GPe population spike trains similarly increased with increasing STN-GPe and GPe-STN connectivity strengths (Figure 2B). The increase in beta power within the STN and GPe was accompanied by an increase in synchronization of the two populations within the beta band. The beta-band coherence of the STN and GPe neural spike trains increased similarly with increased connectivity strengths (Supplementary Figure 1).

Both models exhibited beta-band oscillations as STN-GPe and GPe-STN connectivity increased, indicating that the firing-rate abstraction well captures the oscillatory dynamics of the conductance-based model. Differences between the two models are, however, observed. In particular, in the firing-rate model, the transition from non-oscillatory to oscillatory behavior occurs more abruptly than in the conductance-based model and oscillatory conditions are associated with a relatively stronger coupling from STN to GPe than from GPe to STN. Also, in the conductance-based model, fluctuations in the frequency and amplitude of the beta oscillations are apparent as GPe-STN connectivity increases. Nevertheless, the overall behavior of the models is qualitatively similar with oscillations emerging in both models as STN-GPe connectivity and GPe-STN connectivity increase.

#### 3.1.2. Exogenous Oscillations

**Figure 3:**
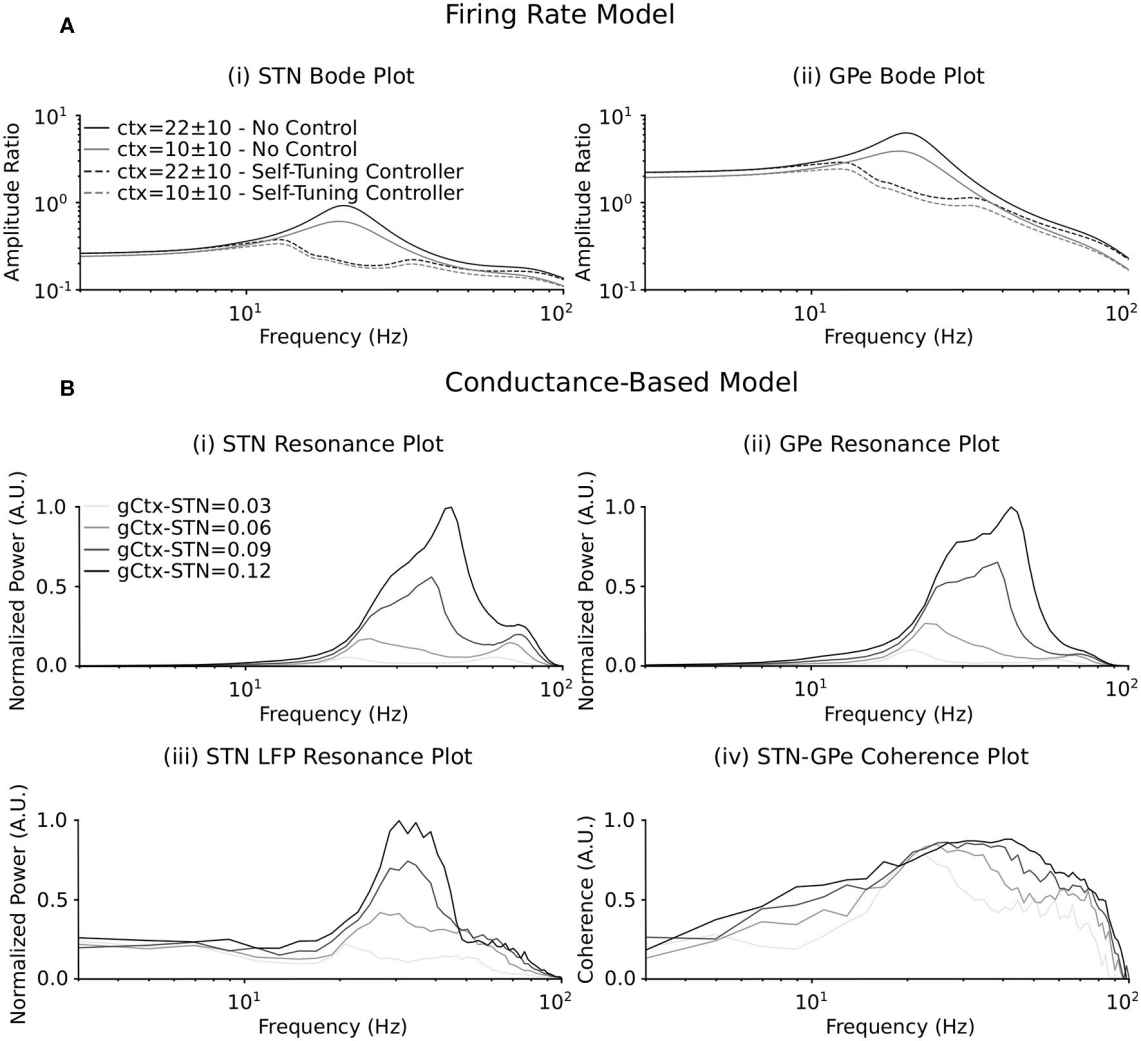
Resonance behavior of the firing rate and conductance-based models in response to synchronous cortical drives. **(A)** Bode plot illustrating amplification of cortical input signals of varying frequency by the (i) STN and (ii) GPe populations in the firing rate model. The amplitude ratio is defined as the amplitude of the input oscillation (the cortical input signal) to the amplitude of the steady state oscillation in the (i) STN and (ii) GPe populations at each cortical input frequency. Solid lines represent the frequency response of each population when DBS is off. Dashed lines represent the frequency response of each population when the self-tuning DBS (7) is implemented with σ = 0.1 and τ_θ_ = 50 ms. Two mean values of the cortical input signal are represented. **(B)** Resonance plot of the power centered at the cortical input frequency in the (i) STN and (ii) GPe cumulative spike trains, (iii) the STN LFP power spectrum and (iv) the coherence between the STN and GPe populations for varying cortical input frequencies. Power at the cortical input frequency was estimated in the cumulative spike trains and LFP power spectra by integrating the power in a 4 Hz window centered on the input frequency in the respective power spectra. The coherence between the STN and GPe populations at the cortical input frequency was estimated from pairs of composite spike trains randomly chosen from the STN and GPe populations (Farina et al., 2014; McManus et al., 2019). The spike trains in the STN and GPe were summed to obtain two composite spike trains. The magnitude squared coherence between the two composite spike trains was then calculated with 1 s overlapping Hamming windows. This was repeated for 200 randomly chosen combinations of spike trains from the STN and GPe populations, as each combination will generate a slightly different coherence estimate. The coherence between the populations was then estimated as the median coherence spectrum over all 200 combinations. The coherence at the cortical input frequency was determined by integrating the resulting coherence spectrum in the 4 Hz window centered on the input frequency. Four cortical to STN connectivity strengths are represented, where the resonance responses are normalized between 0 and 1 in each panel separately.

When synaptic coupling between STN and GPe is sufficiently low, the firing-rate model of the STN-GPe loop is not only stable but also entrainable, meaning that any *T*-periodic input (whether cortical or striatal) generates *T*-periodic steady-state solutions (Chaillet et al., 2013). While this feature is guaranteed for stable linear systems, the nonlinear nature of the firing-rate model makes it less straightforward. This entrainability is a fundamental requirement for constructing frequency profiles of the STN-GPe network. By considering a sinusoidal input at a given frequency, it is indeed possible to measure the magnitude of the resulting steady-state oscillations, and thus the amplification of the network at this specific frequency (Pavlov et al., 2007). Repeating this procedure across a range of input frequencies, we obtained the nonlinear Bode plots depicted as solid lines in Figure 3A.

When DBS is off (solid curves), a clear resonance is observed in the beta frequency band, thus indicating that the network preferably amplifies beta components of the cortical input. This resonance can therefore be interpreted as the beta generation mechanism in the exogenous hypothesis. Due to the nonlinear nature of the firing-rate model, this resonance strength depends on the amplitude of the applied cortical input with more pronounced resonance occurring for stronger mean cortical input.

Akin to the resonance behavior in the firing-rate model, the influence of an external cortical oscillatory input to the STN-GPe network was investigated in the conductance-based model. The connectivity strengths between the STN and GPe populations were selected to lie below the threshold for which the network generated endogenous beta-band oscillations (at 0.11 μS for both), analogous to the entrainable state in the firing-rate model. The frequency of cortical inputs to the STN were varied from 3 to 100 pulses per second, while striatal input to the network remained fixed at 3 pulses per second.

The frequency response of the STN-GPe network due to synchronous cortical inputs through the hyperdirect pathway was examined by estimating the power of the cumulative population spike trains and the spike train coherence between STN and GPe populations within a 4 Hz window centered on the mean frequency of the cortical input. Additionally, resonant network activity was calculated by estimating the power in the simulated STN LFP at the cortical input frequency. Resonance effects were examined as the strength of cortical connectivity to the STN was systematically increased from 0.03 to 0.12 μS (Figure 3B).

As the strength of the hyperdirect pathway was increased, a beta-band resonance emerged in the STN and GPe populations and in the power spectrum of the STN LFP. This was accompanied by synchronization across the STN and GPe populations, as evidenced by a peak in the coherence between STN and GPe spike trains for cortical inputs in the beta frequency range (Figure 3B). Further strengthening of the hyperdirect pathway led to a broadening of the frequency band at which resonance occurred in the cumulative spike trains of the STN and GPe populations and the STN LFP, extending beyond the beta-band. Synchronous cortical inputs to the STN at low connectivity strengths in the beta band and at frequencies outside this range resulted in coherent activity in the subnetwork, but with relatively low power (Figure 3B). Consistent with the firing-rate model (Figure 3A), the beta-band resonance was more pronounced for stronger cortical inputs.

### 3.2. Self-Tuning Controller Assessment

#### 3.2.1. Firing-Rate Model

##### 3.2.1.1. Endogenous oscillations with beta-band activity variation

**Figure 4:**
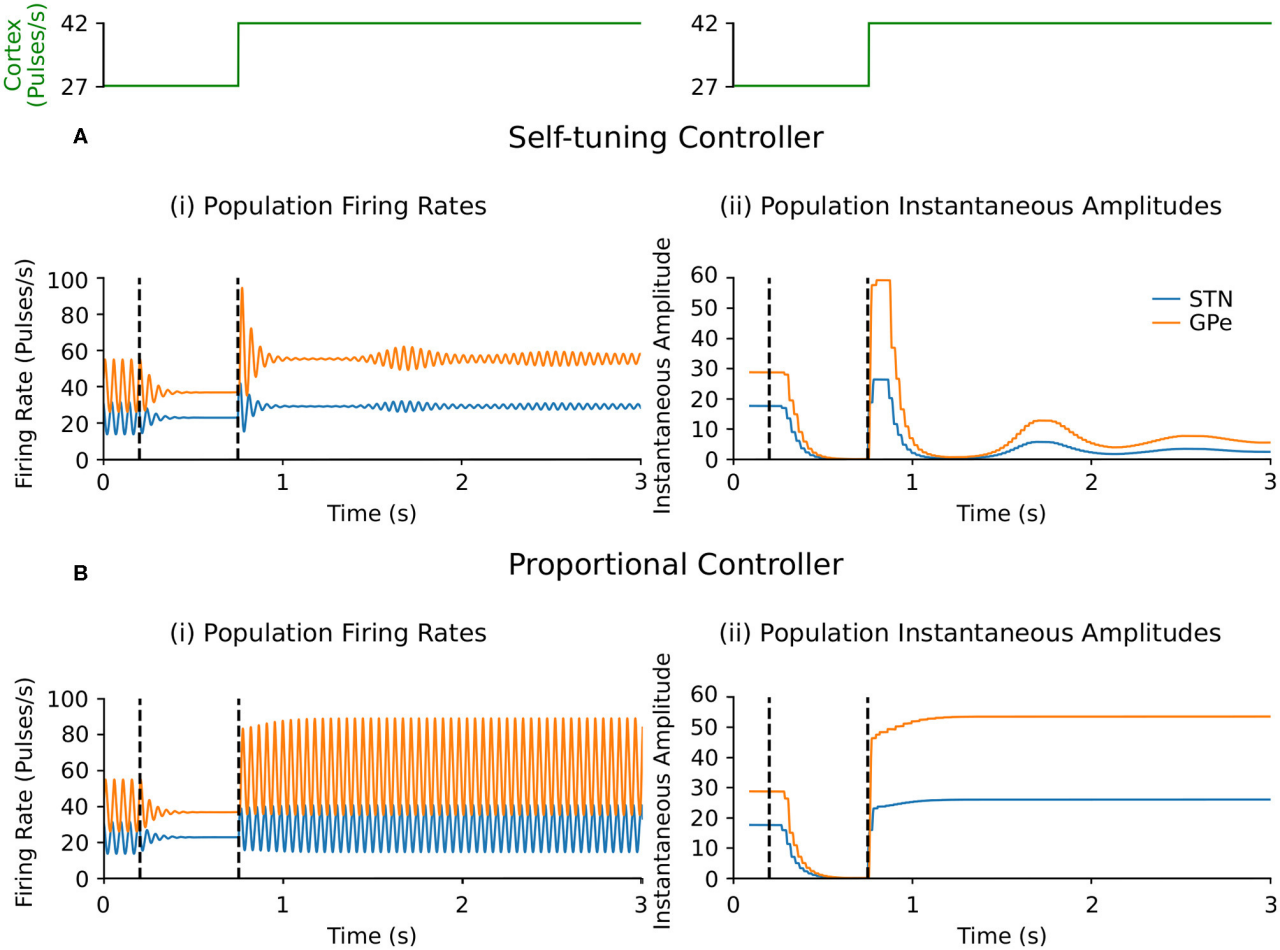
Performance comparison of self-tuning and proportional controllers on endogenous oscillations in the firing-rate model. During simulation, DBS was initially off and then switched on at *t* = 200 ms, where either the **(A)** self-tuning controller or **(B)** proportional controller was implemented. At *t* = 750 ms, the magnitude of the constant cortical input to STN was artificially increased by from 27 to 42, amplifying the endogenous oscillations present in the STN and GPe. The cortical input is presented in green at the top of the figure, aligned with respect to the simulation time with the plots underneath. Left panels illustrate the firing rate behavior of the STN and GPe populations and the right panels correspond to the instantaneous peak-to-peak oscillation amplitude of each population (measured with a sliding window of 100 ms) due to either **(A)** the self-tuning controller (7) with τ_θ_ = 75 ms and σ = 0.19 or **(B)** the proportional controller (4) with θ = 2.

Suppression of endogenous beta oscillations during DBS was assessed first in the firing-rate model, with synaptic weights *c*_12_ and *c*_21_ between STN and GPe increased to generate beta oscillations within the network. A performance comparison between self-tuning DBS (6) and proportional DBS (4) is presented in Figure 4. Both strategies successfully disrupt pathological oscillations between 200 and 750 ms. At *t* = 750 ms, an additional increase of the cortical input to STN was artificially introduced to simulate an increase in beta oscillations. While the self-tuning DBS automatically adapts the proportional gain θ to maintain the attenuation of beta oscillations, pure proportional DBS is unable to do so, resulting in strong beta oscillations that cannot be counteracted without manual tuning of the proportional gain. The self-tuning controller thus outperforms the proportional controller in terms of robustness to disease evolution in the simulated endogenous mechanism scenario.

##### 3.2.1.2. Exogenous oscillations with beta-band activity variation

**Figure 5:**
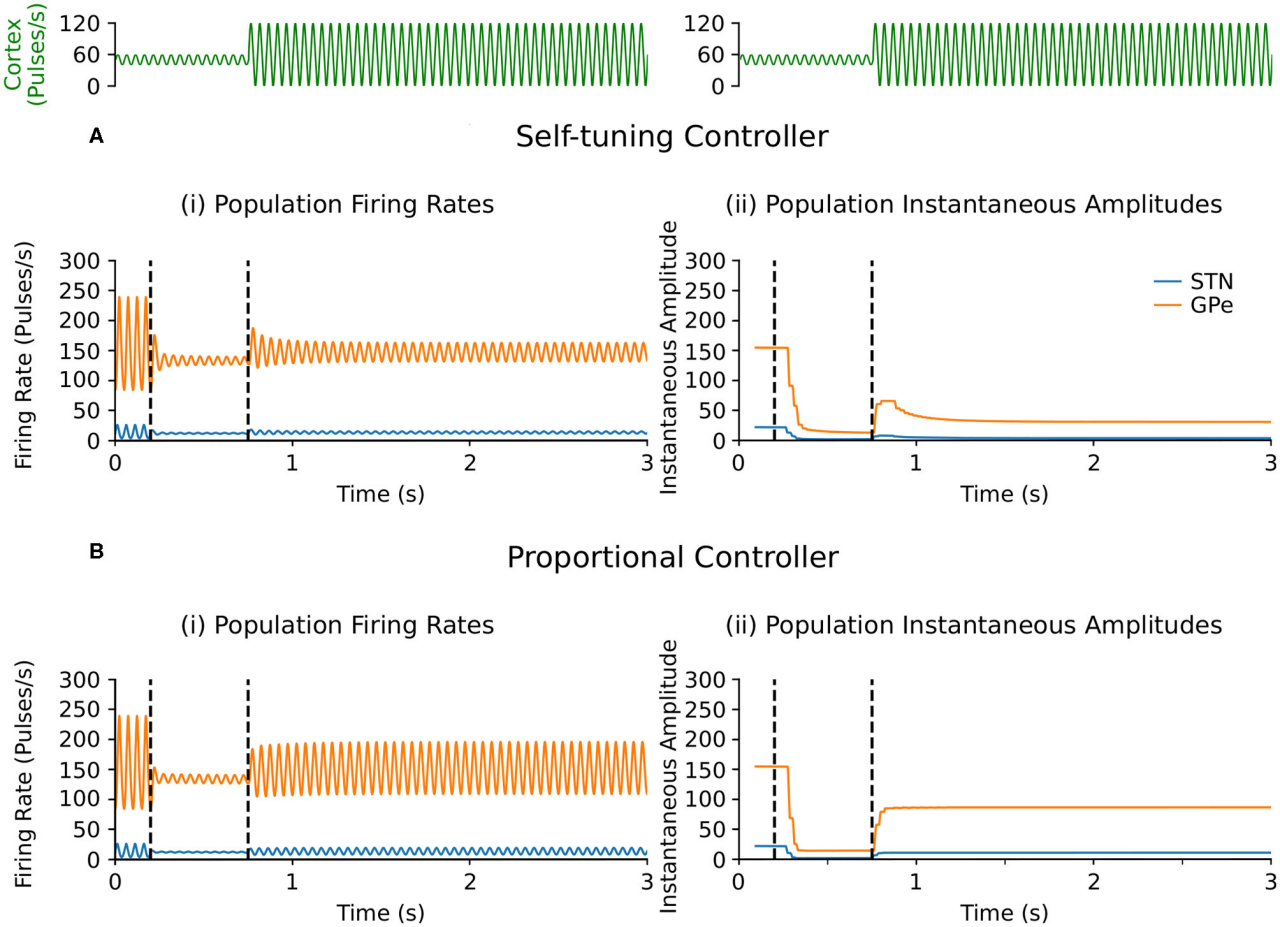
Performance comparison of self-tuning and proportional controllers on exogenous 20 Hz oscillations in the firing-rate model. During simulation, DBS was initially off and then switched on at *t* = 200 ms, where either the **(A)** self-tuning controller or **(B)** proportional controller was implemented. At *t* = 750 ms, the amplitude of the cortical oscillations was increased from 10 to 60 and the mean level of the oscillations is raised from 50 to 60, amplifying the exogenous oscillations present in the STN and GPe. The cortical input is presented in green at the top of the figure, aligned with respect to the simulation time with the plots underneath. Left panels illustrate the firing rate behavior of the STN and GPe populations and the right panels correspond to the instantaneous peak-to-peak oscillation amplitude of each population (measured with a sliding window of 100 ms) due to either **(A)** the self-tuning controller (7) with τ_θ_ = 5 ms and σ = 0.01 or **(B)** the proportional controller (4) with θ = 25.

Selective disruption of pathological beta oscillations generated through exogenous inputs to the STN using the self-tuning controller (7) was then confirmed. With the self-tuning DBS (dashed curves) (Figure 3A) the beta-band resonance was eliminated in the firing-rate model, while the frequency profile of the STN-GPe network remained essentially unaltered in other frequency bands, as DBS remains off when beta activity is not detected within the STN. Figure 5 illustrates that, similar to the endogenous case, self-tuning DBS (6) outperforms proportional DBS (4) when faced with changes in the exogenous oscillations. After the stimulation is turned on at *t* = 200 ms, both controllers successfully decrease the amplitude of the pathological oscillations. After the mean level of the cortical input as well as the amplitude of oscillations is increased at *t* = 750 ms, the self-tuning controller achieves higher damping of the oscillations than the proportional controller.

#### 3.2.2. Conductance-Based Model

##### 3.2.2.1. Scenario 1: beta-band target variation

**Figure 6:**
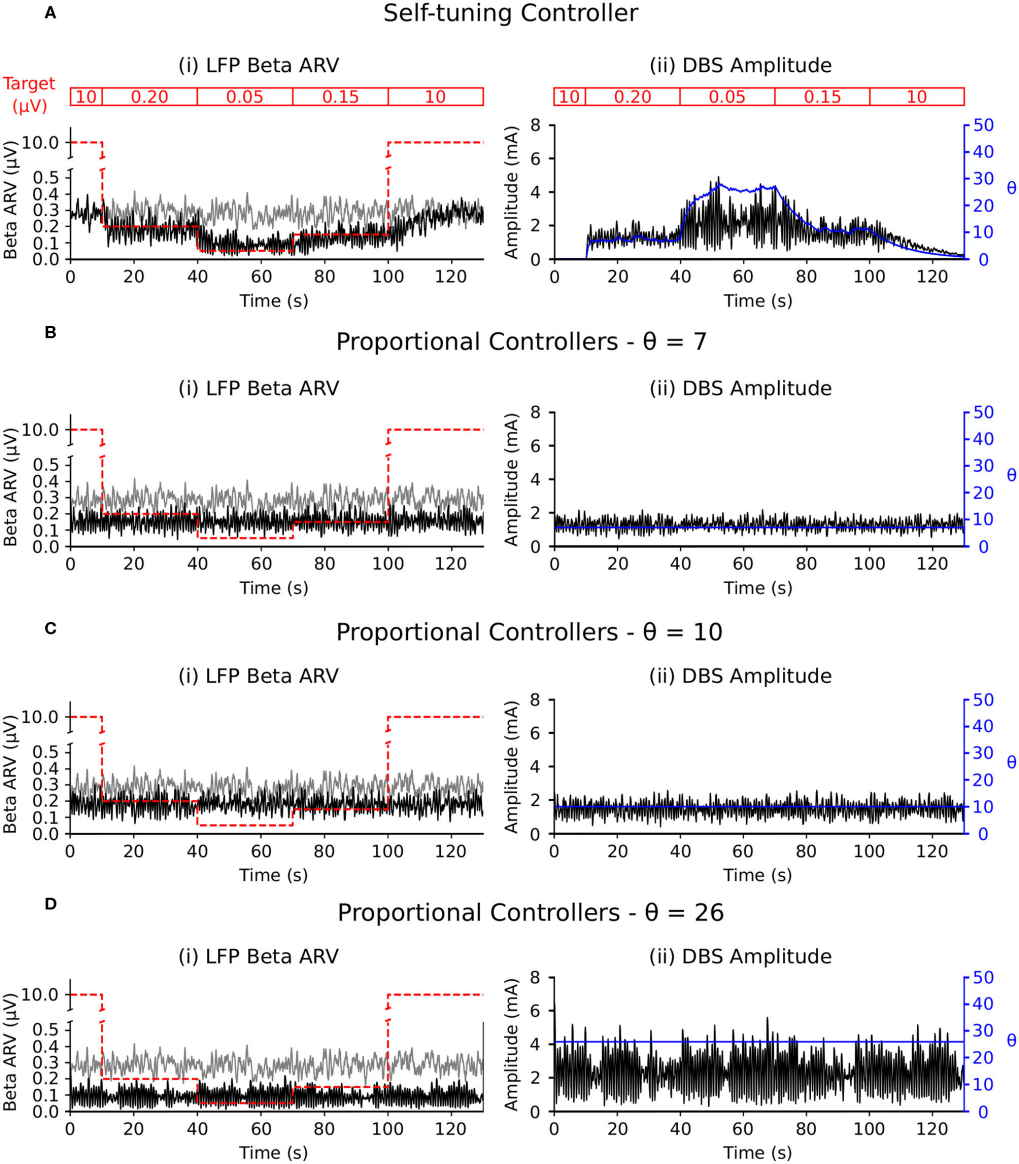
Performance comparison of self-tuning and proportional controllers in response to beta ARV target variations in the conductance-based model. Background beta-band activity in the model was fixed to its maximum value for the duration of the simulation while the target value was modulated to 10, 0.20, 0.05, 0.15, and 10 μV during the 0–10, 10–40, 40–70, 70–100, and 100–130 s time periods, respectively. The target value for each time period is highlighted by the red segmented bar at the top of the figure. Left panels illustrate the target value (dashed red), the beta ARV measured from the STN LFP when DBS was off (gray) or on (black), where the DBS amplitude is modulated by the corresponding controller. Right panels illustrate the time evolution of the DBS amplitude (black) and controller gain θ (blue) during simulation. **(A)** Self-tuning DBS controller with τ_θ_ = 100 ms and σ = 0.00875. **(B)** Proportional controller with a fixed gain value of θ = 7. **(C)** Proportional controller with a fixed gain value of θ = 10. **(D)** Proportional controller with a fixed gain value of θ = 26.

The self-tuning controller maintained the LFP beta ARV around the desired target values between 10 and 100 s, and turned off from 0 to 10 and 100 to 130 s, where the target value was high and stimulation was not required (Figure 6A). The self-tuning controller resulted in a mean power consumption of 11.0 μW and a 82.1% reduction in the MSE. Proportional controllers with fixed gain were able to maintain beta ARV at a single target value, however they were unsuitable for target values other than the one for which they were tuned and resulted in either under or over stimulation when attempting to maintain beta ARV (Figures 6B–D). Proportional controllers with fixed gains at 7, 10 and 26 resulted in mean power consumption values of 6.9, 9.3, and 28.2 μW and reductions in the MSE of 79.4, 85.1, and 81.1%, respectively.

##### 3.2.2.2. Scenario 2: beta-band activity variation

**Figure 7:**
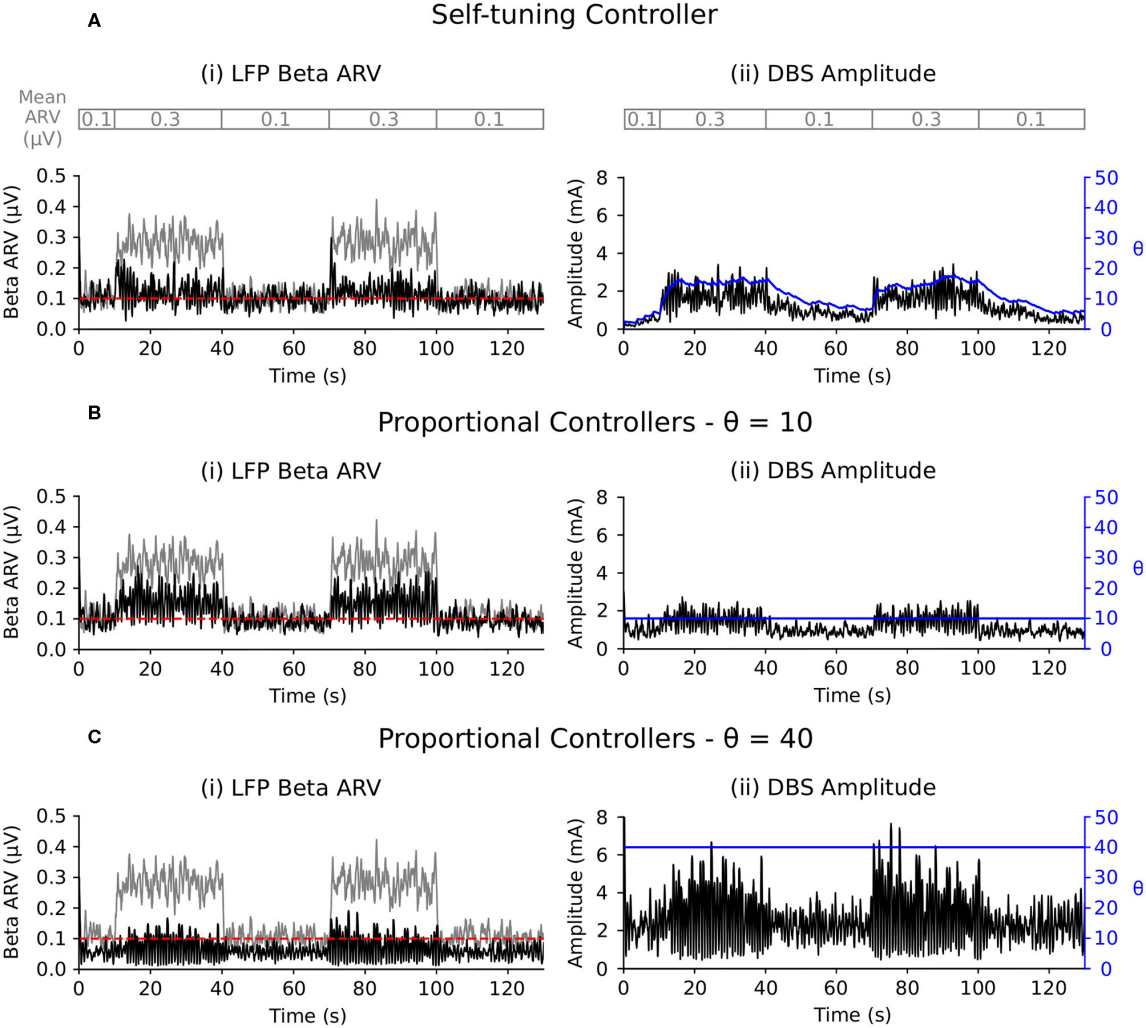
Performance comparison of self-tuning and proportional controllers in response to background beta-band activity variations in the conductance-based model. The beta ARV target value was fixed to 0.1μV for the duration of the simulation while the background beta-band activity was modulated to be at its maximum value during the 10–40 and 70–100 s time periods and at its minimum value during the 0–10, 40–70, and 100–130 s time periods, respectively. The mean ARV of the background beta-band activity measured in the LFP when DBS was off is highlighted by the gray segmented bar at the top of the figure. Left panels illustrate the target value (dashed red), the beta ARV measured from the STN LFP when DBS was off (gray) or on (black), where the DBS amplitude was modulated by the corresponding controller. Right panels illustrate the time evolution of the DBS amplitude (black) and controller gain θ (blue) during simulation. **(A)** Self-tuning DBS controller with τ_θ_ = 100 ms and σ = 0.00875. **(B)** Proportional controller with a fixed gain value of θ = 10. **(C)** Proportional controller with a fixed gain value of θ = 40.

The LFP beta ARV was maintained at the target value by the self-tuning controller as the background beta activity was varied between low and high activity periods (Figure 7A). The self-tuning controller consumed 17.9 μW and resulted in a 91.4 % reduction of the MSE. The proportional controller with a fixed gain value of 10 was able to maintain the LFP beta ARV at the target value during low background beta activity periods, but did not provide sufficient stimulation to suppress to the target value during periods of high background beta activity (Figure 7B). The proportional controller with a fixed gain value of 40 maintained the LFP beta ARV at the target value during both low and high background beta activity periods (Figure 7C). The proportional controllers with fixed gain values of 10 and 40 resulted in MSE reductions of 73.4 and 95.0 % and power consumption values of 6.6 and 34.8 μW, respectively. When maintaining the LFP beta activity at the target value, the proportional controller with a fixed gain value of 40 resulted in over stimulation during the low background activity periods, consuming more power than necessary to maintain the LFP beta ARV.

##### 3.2.2.3. Scenario 3: electrode impedance variation

**Figure 8:**
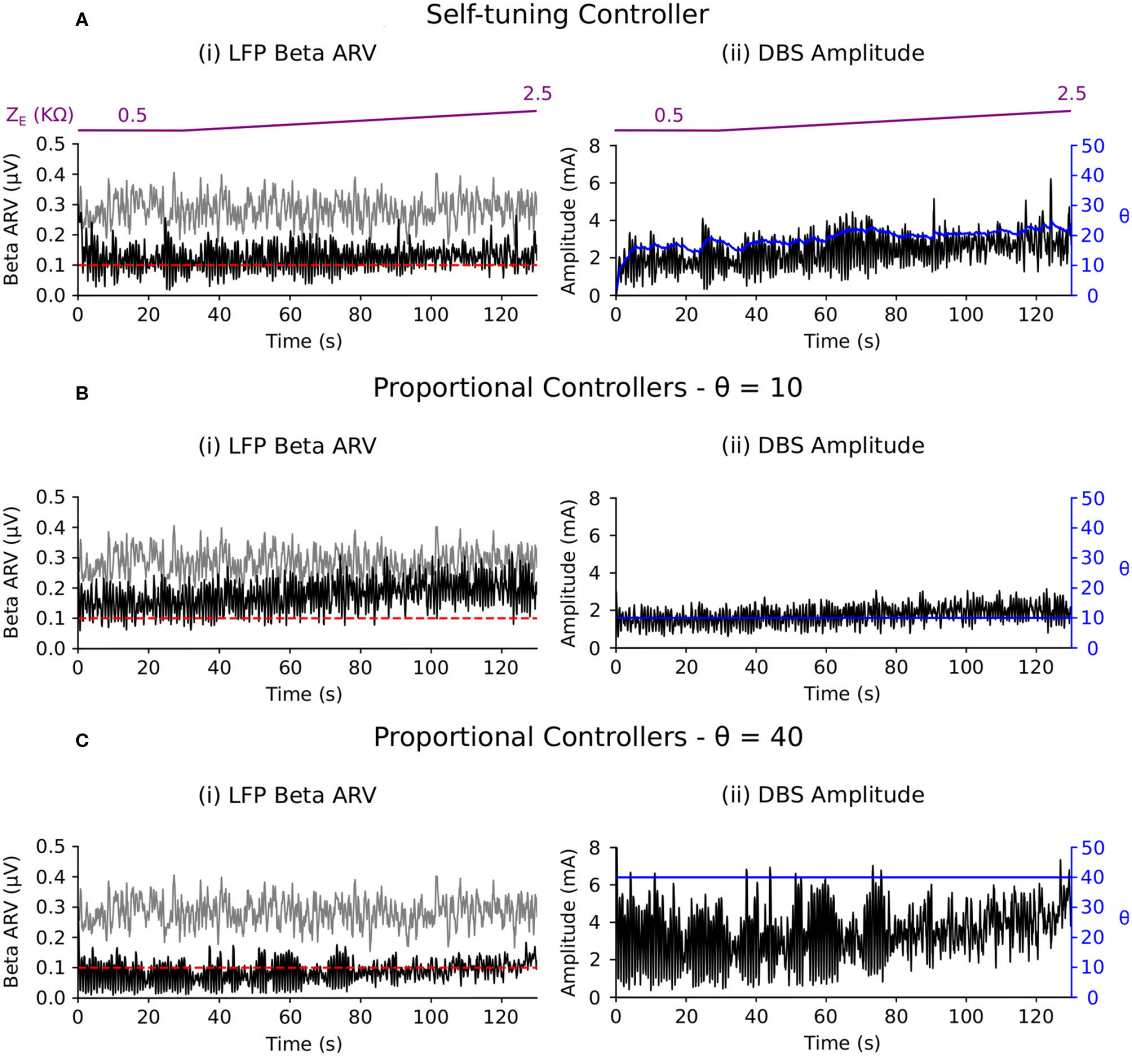
Performance comparison of self-tuning and proportional controllers in response to electrode impedance variations in the conductance-based model. The beta ARV target value was fixed to 0.1μV and the background beta-band activity was fixed to its maximum value for the duration of the simulation. The electrode impedance was fixed at 0.5 KΩ during the 0–30 s period and was then linearly increased from 0.5 to 2.5 KΩ over the 30–130 simulation period. The electrode impedance variation is illustrated by the purple bar at the top of the figure. Left panels illustrate the target value (dashed red), the beta ARV measured from the STN LFP when DBS was off (gray) or on (black), where the DBS amplitude was modulated by the corresponding controller. Right panels illustrate the time evolution of the DBS amplitude (black) and controller gain θ (blue) during simulation. **(A)** Self-tuning DBS controller with τ_θ_ = 100 ms and σ = 0.00875. **(B)** Proportional controller with a fixed gain value of θ = 10. **(C)** Proportional controller with a fixed gain value of θ = 40.

The self-tuning controller maintained the LFP beta ARV at the target level as the electrode impedance was linearly increased over the course of the simulation to five times its initial impedance value, i.e., from an initial value of 0.5–2.5 kΩ (Figure 8A). Background beta activity and the target value of 0.1 μV remained fixed over the course of the simulation. The self-tuning controller resulted in a MSE reduction of 93.5% and a power consumption of 11.2 μW. Proportional controllers with fixed gain values of 10 and 40 lead to MSE reductions of 77.0 and 96.0 % while consuming 6.2 and 27.7 μW, respectively. Similar to scenarios 1 and 2, the self-tuning controller was able to tune its gain to the required level to maintain beta ARV at the target level. Proportional control with fixed gain of 10 became less effective over the course of the simulation, while proportional control with fixed gain of 40 was effective throughout the simulation, but consumed more power than necessary (Figures 8B,C). A summary of the controller performance under the different scenarios considered is presented in Table 2.

**Table 2:** Summary of controller performance in the three considered scenarios.

**Target variation:**	**MSE (**%**)**	**Power consumed (μW)**
DBS Off	100	0
Self-tuning controller	7.9	11.0
Proportional controller (θ = 7)	20.6	6.9
Proportional controller (θ = 10)	14.9	9.3
Proportional controller (θ = 26)	18.9	28.2
**Beta variation:**	**MSE (**%**)**	**Power consumed (μW)**
DBS Off	100	0
Self-tuning controller	8.6	17.9
Proportional controller (θ = 10)	26.6	6.6
Proportional controller (θ = 40)	5.0	34.8
**Electrode impedance variation:**	**MSE (**%**)**	**Power consumed (μW)**
DBS Off	100	0
Self-tuning controller	6.5	11.2
Proportional controller (θ = 10)	23.0	6.2
Proportional controller (θ = 40)	4.0	27.7

*In each scenario, the MSE value is reported as a percentage of the DBS Off case, where the DBS Off case corresponds to a 100% MSE*.

## 4. Discussion

### 4.1. Firing-Rate and Conductance-Based Models

The proposed firing-rate model facilitated the derivation of a robust control law capable of disrupting pathological beta-band oscillations in the STN-GPe network in Parkinson's disease and the analytic establishment of its efficiency. Firing-rate models capture the average behavior of neural populations and facilitate tractable mathematical analysis of network behavior (Destexhe and Sejnowski, 2009). These models, however, summarize the neuronal population to a single variable: the number of spikes it emits per unit time. They are thus unable to capture cellular-level features such as sub-threshold activity, LFP activity, specific responses induced by bursting, antidromic activation of STN afferent inputs or the interaction between neurons and the induced extracellular potential. In contrast, networks of conductance-based neuron models offer the ability to capture more complex network dynamics and interactions, but are usually too complex to analyze mathematically.

While recent approaches have attempted to bridge the gap between these two types of models (di Volo et al., 2019), we have empirically assessed the similarities and discrepancies between them and shown that key features needed to analyze and control pathological oscillations in Parkinson's disease are indeed captured by both models (Figures 2, 3). We also demonstrated in section 2.3 the possibility to check the theoretical stabilization condition (3), derived on the firing-rate model, using observations from the conductance-based model. By utilizing both modeling approaches, the limitations of each model are complemented by the strengths of the other. The joint analysis of these two models also supports the relevance of abstract firing-rate models for the derivation of advanced DBS strategies aiming at counteracting a targeted brain oscillation, which can then be validated in computationally detailed models before preclinical investigations.

### 4.2. Physiological Interpretation of the Stabilizability Condition

The theoretical condition obtained on the firing-rate model to ensure stabilizability by the self-tuning DBS signal reads ℓ_2_*c*_22_ < 1 (see Proposition). In other words, the synaptic weights from GPe to itself should be sufficiently low. This condition ensures that the GPe does not act as a pacemaker on its own, as low internal coupling is a standard sufficient condition for stability of a neuronal population (see for instance Faye and Faugeras, 2010). The necessity to impose that GPe does not generate pathological oscillations on its own is quite reasonable: considering the extreme case when STN is not connected to the GPe, it would be impossible to attenuate self-generated GPe oscillations by stimulating STN only.

It is worth noting that the proposed condition precludes GPe self-oscillatory activity no matter the value of its internal delay. This constitutes a noteworthy feature of our mathematical result as this delay does not need to be estimated. Nevertheless, for realistic values of internal GPe delays (of the order of few milliseconds), no such self-oscillatory GPe activity is observed even if the condition is violated, at least for reasonable values of the striatal input. This was confirmed in numerical simulations of the conductance-based model in which the internal GPe synaptic weights were artificially increased by two orders of magnitude. Even in that case, GPe was unable to autonomously generate oscillations and the proposed self-tuning DBS successfully disrupted network beta oscillations (data not shown).

### 4.3. Endogenous and Exogenous Generation of Oscillations

Beyond employing two modeling approaches of the structures involved, the paper also investigated two possible mechanisms of pathological oscillations generation: the emergence of endogenous oscillations, in which the STN-GPe network acts as a pacemaker, and the generation of oscillations through the interaction of the network with inputs originating from other structures such as the cortex.

The ability of the STN-GPe network to endogenously generate beta-band oscillations was consistent across both the firing-rate and conductance-based models (Figure 2). The STN-GPe network has been proposed as a potential source of pathologically increased beta-band oscillations in Parkinson's disease, where connectivity changes in the reciprocally connected network leads to the endogenous generation of beta-band oscillations. This behavior has been previously explored in modeling studies utilizing firing-rate models where the progression of Parkinson's disease is represented as an increase in the synaptic coupling strengths between the STN and GPe neuron populations (Nevado-Holgado et al., 2010; Pavlides et al., 2012; Pasillas-Lépine, 2013). The firing-rate model presented here is consistent with these previous studies in which a Hopf bifurcation occurs and leads to beta-band oscillations in the network when the synaptic coupling strengths are sufficiently increased (Figure 2A). This behavior is well-matched in the conductance model, where increases in the synaptic coupling strengths between the two populations also leads to the endogenous emergence of beta-band oscillations in the network (Figure 2B).

Although modeling studies support the hypothesis that the reciprocally connected STN-GPe network is capable of generating the beta-band oscillatory activity, investigations of isolated STN-GPe cell cultures *in vitro* have observed the emergence of endogenous oscillations at much lower frequencies (Plenz and Kital, 1999). Furthermore, increasing evidence from experimental studies in patients and animal models suggest that external inputs to the STN-GPe loop may play a key role in the generation of elevated beta-band oscillations in the parkinsonian cortex and basal ganglia. The STN-GPe network occupies a crucial location in the cortical basal ganglia network receiving inputs from both cortical and striatal structures through the hyperdirect and indirect pathways, respectively. Due to connectivity changes in the STN-GPe loop during Parkinson's disease, it is thus hypothesized that exogenous beta oscillations are locally amplified by the loop, with subsequent oscillations then propagating throughout the full cortical basal-ganglia network (Magill et al., 2001; Mallet et al., 2008; Corbit et al., 2016; West et al., 2018). Consistent with this, Sharott et al. (2005) observed that the power and coherence of beta oscillations in the cortex and STN were elevated during dopamine depletion in a parkinsonian rat model, while Litvak et al. (2011) observed cortical beta activity leading to STN beta activity in parkinsonian patients. In primate studies, oscillatory activity in the GPe was observed to be mainly due to excitatory activity from the STN, while oscillatory activity in the STN was primarily due to excitatory cortical input (Tachibana et al., 2008, 2011).

Both the firing-rate and conductance-based models in the present study demonstrated the STN-GPe network's ability to entrain to an external cortical rhythm (Figure 3). A Bode plot of the input-output relationship in the firing-rate model showed a marked peak in the beta frequency band, with this peak increasing in magnitude and broadening with increasing strength of the inputs to the STN (Figure 3A). This behavior was consistent with the conductance-based model behavior, where the STN-GPe network displayed a resonant peak in the beta frequency band for low connectivity strength between the cortex and STN (Figure 3B). Increasing the strength of the hyperdirect pathway led to increased resonance in the model and also to a widening of the frequency bands where resonance was observed (Figure 3B). This observed resonance is consistent with other modeling investigations of the STN-GPe network, where firing-rate models (Nevado-Holgado et al., 2014; Detorakis and Chaillet, 2017; Liu et al., 2017, 2020) and conductance-based neuron models (Ahn et al., 2016; Shouno et al., 2017; Koelman and Lowery, 2019) were used to investigate the behavior of STN-GPe network in response to external drives. Although those studies have illustrated the resonant capabilities of the STN-GPe network of both firing-rate and conductance-based models separately, this study is the first to demonstrate that both modeling approaches lead to comparable results (Figure 3).

### 4.4. Self-Tuning DBS Controller

Having established qualitative consistency in the behavior of the firing-rate and conductance-based models, the performance of the proposed self-tuning DBS controller was first proven mathematically and validated in the firing-rate model where the controller was capable of disrupting both exogenously and endogenously generated beta-band activity in the STN-GPe network (Figures 3A, 4, 5). The simulations confirmed that the self-tuning controller autonomously adapts its gain value to the minimal value required to counteract pathological oscillations, thus avoiding over-stimulation and allowing for adaptation to possible changes in the system properties associated with disease progression (Figures 4, 5).

The performance of the controller to maintain network beta-band oscillations at a target level was then assessed in the conductance-based model in three example conditions, which emulated practical situations in which gain adaptation may be required *in vivo*: modification of the beta-level target, variation of the beta oscillations intensity, and alteration of the electrode impedance.

#### 4.4.1. Adaptation to Target-Level Changes

The self-tuning controller adapted the controller gain in response to changes in the target value (Figure 6A). For each target value, the controller identified the necessary gain to maintain the biomarker at the target level. In contrast, proportional controllers with fixed gain were unable to track target changes and resulted in less reduction in the MSE than the self-tuning controller (Figures 6B–D, Table 2). The self-tuning controller consumed more power than the controllers with low fixed gain and less power than the controller with high fixed gain, but was able to maintain low error as the target changed (Table 2).

Similar issues were identified by Su et al. (2019) who investigated the ability of a proportional-integral controller to modulate DBS frequency to track dynamic changes in a target signal during closed-loop DBS using a conductance-based model. While the proportional-integral controller considered in that study was able to successfully track dynamic changes in the target beta signal, it required different controller gain values for each beta-band target level considered. The adaptive controller proposed here overcomes this issue as the controller self-tunes its gain value to find the gain necessary for maintaining the beta ARV at the target values (Figure 6A).

#### 4.4.2. Adaptation to Beta Oscillation Fluctuations

The self-tuning controller was able to maintain the biomarker at the target level while beta activity in the network varied (Figure 7A). Between *t* = 0 and *t* = 10 s, the self-tuning controller adapted its gain value to the low beta activity. Once beta activity in the network increased, during the 10–40 s period, the controller increased its gain to suppress the beta activity to the target level. The proportional controller with low fixed gain value (θ = 10) was able to maintain the biomarker at the target level during low beta activity periods, but was unable to during high beta periods (Figure 7B). In contrast, the proportional controller with a high fixed gain value (θ = 40) was able to maintain the biomarker close to the target during both low and high beta activity periods (Figure 7C), but at the cost of increased power consumption (Table 2).

In line with the power consumption in scenario 1, the controllers with low and high fixed gain resulted in the lowest and highest power consumption, respectively, and the power consumption of the self-tuning controller lay between these values (Table 2). Essentially, the self-tuning controller identified the gain required to maintain the beta ARV at the target level in both the high and low beta activity periods and consumed the necessary power to maintain beta at the target.

Clinical investigations of proportional control strategies in patients with Parkinson's disease (Rosa et al., 2015; Arlotti et al., 2018), have observed attenuation of DBS when subjects were on and off medication, motivating the need for self-tuning in response to varying background beta activity. Arlotti et al. (2018) investigated proportional control over an 8 h period and showed a 30–45 % improvement in patient UPDRS III motor scores. Although these clinical studies showed promising results, we hypothesize that additional benefits may result from using the self-tuning controller proposed here. The proportional control scheme implemented in Rosa et al. (2015) and Arlotti et al. (2018) utilized a fixed gain value, and thus may lead to under or over stimulation if a suitable gain is not selected.

#### 4.4.3. Adaptation to Electrode Impedance Variations

Clinical measurements of electrode impedance usually vary between 0.5 and 1.5 kΩ (Obeso et al., 2001; Volkmann et al., 2002). However, many factors contribute to the electrode impedance, and its variability between subjects. These factors include the surface properties of the electrodes, electrical double layer, conductivity of the bulk tissue medium and thickness of the surrounding encapsulation layer (Butson et al., 2006). The electrode impedance plays a crucial role in determining the current delivered to tissue during voltage-controlled stimulation. Clinical investigations of closed-loop DBS have not yet investigated the impact of electrode impedance variations on DBS efficiency as the timescales at which these variations take place are much longer than the current timescales at which closed-loop DBS has been investigated clinically. For closed-loop DBS to remain effective when chronically implemented, it is necessary to utilize controllers which can accommodate such changes and avoid a need for clinical retuning of the controller parameters. This third scenario, therefore, aimed to assess this robustness to electrode impedance variations. In response to the simulated changes, the self-tuning controller gradually increased the controller gain to maintain the biomarker at the target level (Figure 8A).

In contrast, the proportional controller with low fixed gain was able to maintain the beta ARV at the target up until *t* = 60 s, but became less effective thereafter (Figure 8B). With a higher fixed gain value, the proportional controller was able to accommodate changes in the electrode impedance and remained effective at suppressing the beta ARV to the target for whole the duration of the simulation (Figure 8C).

In line with the other examples, the power consumption of the self-tuning controller was greater than that of the proportional controller with low fixed gain and less than the proportional controller with the high fixed gain (Table 2). Although both the proportional controller with high fixed gain and the self-tuning controller successfully maintained beta activity at its target level, more power consumption was needed for the former than for the latter.

### 4.5. Clinical Implementation of the Self-Tuning DBS

The self-tuning controller presented here offers several advantages for experimental implementation. First, it relies only on data from the STN, no additional recording electrodes in other brain structures are required. Moreover, although the firing-rate model utilized the number of STN pulses per second, the simulations conducted in the conductance-based model showed the efficiency of the approach when only STN LFP is accessible. More importantly, the self-tuning controller relies on very limited information regarding the system parameters. The theoretical result of Proposition simply requires that the GPe internal synaptic weights are sufficiently low. This condition, also present in theoretical investigations on fixed-gain proportional DBS (Chaillet et al., 2017), does not require precise knowledge on the exact shape of activation functions, or the values of the time constants, synaptic weights, or delays.

Nonetheless, experimental validation of the self-tuning controller also comes with challenges. First, both the stimulation and the recording are assumed to be within the STN. This would lead to stimulation artifacts that should be removed. Several techniques are available to address this issue (Rossi et al., 2007; Stanslaski et al., 2012; Basir-Kazeruni, 2017) The proposed controller also requires some embedded computational capabilities in order to filter the STN LFP and implement the control law (7). While more demanding than classical open-loop DBS, the required computational power is comparable to the algorithms employed for proportional or on-off stimulation. In addition, the upper and lower bounds on the applied current or voltage would be the same as those used during conventional stimulus parameter setting.

Despite its self-tuning nature, the control law (7) requires two parameters to be chosen: the time constant τ_θ_ and the dissipation parameter σ. Both these parameters have a strong impact on the beta attenuation performance. For the sake of illustration, these parameters have been chosen here to demonstrate quick adaptation of the gain over the simulation duration. In an experimental setting, τ_θ_ should be chosen on the order of the timescale at which the targeted variations occur. Generally speaking, a large value of τ_θ_ is more convenient for adaptation to slow variations. σ should be chosen as a compromise between rapid decrease of the gain when the beta level is low (high σ) and limited gain overshoot in response to an increase of the beta level (low σ). In practice, the controller parameters could also be obtained through an optimization process, such as the dual-loop framework outlined in Grado et al. (2018), though an optimization approach would itself also require the identification of appropriate objective functions which may be non-trivial.

In summary, the proposed self-tuning DBS controller offers increased robustness to variations in system properties including changes in the strength of beta oscillations and or the electrode-tissue interface, and can accommodate alterations in the desired beta level. Compared to fixed-gain proportional control, it provides a better compromise between power consumption and efficient attenuation of beta oscillations, with little additional computational complexity or technological requirements.

## Data Availability Statement

The models presented in this study can be found in online repositories. The names of the repository/repositories and accession number(s) can be found below: The firing-rate model is available to download from GitHub (https://github.com/silvanx/self-tuning-control). The conductance-based model is available to download from ModelDB (https://senselab.med.yale.edu/modeldb/) at ascension number 262046.

## Author Contributions

JO has developed and implemented the firing-rate model. JF has developed and implemented the conductance-based model. JO and AC have established the Proposition in Section 2.1.1. AC and ML have coordinated and supervised the study. All authors have contributed to the paper writing.

## Conflict of Interest

The authors declare that the research was conducted in the absence of any commercial or financial relationships that could be construed as a potential conflict of interest.

## References

[B1] Ahn S. Zauber S. E. Worth R. M. Rubchinsky L. L. (2016). Synchronized beta-band oscillations in a model of the globus pallidus-subthalamic nucleus network under external input. Front. Comput. Neurosci. 10:134. 10.3389/fncom.2016.00134 28066222 PMC5167737

[B2] Arlotti M. Marceglia S. Foffani G. Volkmann J. Lozano A. M. Moro E.. (2018). Eight-hours adaptive deep brain stimulation in patients with Parkinson disease. Neurology 90, e971–e976. 10.1212/WNL.0000000000005121 29444973 PMC5858949

[B3] Basir-Kazeruni S. (2017). Energy-efficient DSP solutions for simultaneous neural recording and stimulation (Ph.D. thesis). ProQuest Dissertations Publishing, Ann Arbor, MI.

[B4] Beuter A. Lefaucheur J.-P. Modolo J. (2014). Closed-loop cortical neuromodulation in Parkinson's disease: an alternative to deep brain stimulation? Clin. Neurophysiol. 125, 874–885. 10.1016/j.clinph.2014.01.006 24555921

[B5] Butson C. R. Maks C. B. McIntyre C. C. (2006). Sources and effects of electrode impedance during deep brain stimulation. Clin. Neurophysiol. 117, 447–454. 10.1016/j.clinph.2005.10.007 16376143 PMC3692979

[B6] Carron R. Chaillet A. Filipchuk A. Pasillas-Lepine W. Hammond C. (2013). Closing the loop of deep brain stimulation. Front. Syst. Neurosci. 7:112. 10.3389/fnsys.2013.00112 24391555 PMC3868949

[B7] Chaillet A. Detorakis G. I. Palfi S. Senova S. (2017). Robust stabilization of delayed neural fields with partial measurement and actuation. Automatica 83, 262–274. 10.1016/j.automatica.2017.05.011

[B8] Chaillet A. Orłowski J. Pepe P. (2019). “A relaxed Lyapunov-Krasovskii condition for global exponential stability of Lipschitz time-delay systems,” in 58th IEEE Conference on Decision and Control (CDC) (Nice).

[B9] Chaillet A. Pogromsky A. Rüffer B. (2013). “A Razumikhin approach for the incremental stability of delayed nonlinear systems,” in 52nd IEEE Conference on Decision and Control (CDC) (Florence).

[B10] Corbit V. L. Whalen T. C. Zitelli K. T. Crilly S. Y. Rubin J. E. Gittis A. H. (2016). Pallidostriatal projections promote β oscillations in a dopamine-depleted biophysical network model. J. Neurosci. 36, 5556–5571. 10.1523/JNEUROSCI.0339-16.2016 27194335 PMC4871989

[B11] Davidson C. M. de Paor A. M. Cagnan H. Lowery M. M. (2016). Analysis of oscillatory neural activity in series network models of Parkinson's disease during deep brain stimulation. IEEE Trans. Biomed. Eng. 63, 86–96. 10.1109/TBME.2015.2475166 26340768

[B12] Davison A. P. Brüderle D. Eppler J. M. Kremkow J. Muller E. Pecevski D.. (2009). PyNN: a common interface for neuronal network simulators. Front. Neuroinformatics 2:11. 10.3389/neuro.11.011.2008 19194529 PMC2634533

[B13] Destexhe A. Mainen Z. Sejnowski T. (1994). An efficient method for computing synaptic conductances based on a kinetic model of receptor binding. Neural Comput. 6, 14–18. 10.1162/neco.1994.6.1.14

[B14] Destexhe A. Sejnowski T. J. (2009). The Wilson–Cowan model, 36 years later. Biol. Cybern. 101, 1–2. 10.1007/s00422-009-0328-3 19662434 PMC2866289

[B15] Detorakis G. I. Chaillet A. (2017). “Incremental stability of spatiotemporal delayed dynamics and application to neural fields,” in 56th IEEE Conference on Decision and Control (CDC) (Melbourne, QLD).

[B16] di Volo M. Romagnoni A. Capone C. Destexhe A. (2019). Biologically realistic mean-field models of conductance-based networks of spiking neurons with adaptation. Neural Comput. 31, 653–680. 10.1162/neco_a_01173 30764741

[B17] Eitan R. Bergman H. Israel Z. (2019). “Closed-loop deep brain stimulation for Parkinson's disease,” in Surgery for Parkinson's Disease, ed R. R. Goodman (Cham: Springer), 131–149.

[B18] Eusebio A. Thevathasan W. Doyle G. L. Pogosyan A. Bye E. Foltynie T.. (2011). Deep brain stimulation can suppress pathological synchronisation in parkinsonian patients. J. Neurol. Neurosurg. Psychiatry 82, 569–573. 10.1136/jnnp.2010.217489 20935326 PMC3072048

[B19] Farina D. Negro F. Dideriksen J. (2014). The effective neural drive to muscles is the common synaptic input to motor neurons. J. Physiol. 592, 3427–3441. 10.1113/jphysiol.2014.273581 24860172 PMC4229341

[B20] Faye G. Faugeras O. (2010). Some theoretical and numerical results for delayed neural field equations. Phys. D 239, 561–578. 10.1016/j.physd.2010.01.010

[B21] Fleming J. Dunn E. Lowery M. (2020). Simulation of closed-loop deep brain stimulation control schemes for suppression of pathological beta oscillations in Parkinson's disease. Front. Neurosci. 14:166. 10.3389/fnins.2020.00166 32194372 PMC7066305

[B22] Foust A. Yu Y. Popovic M. Zecevic D. McCormick D. (2011). Somatic membrane potential and kv1 channels control spike repolarization in cortical axon collaterals and presynaptic boutons. J. Neurosci. 31, 15490–15498. 10.1523/JNEUROSCI.2752-11.2011 22031895 PMC3225031

[B23] Fradkov A. L. Miroshnik I. V. Nikiforov V. O. (1999). Nonlinear and Adaptive Control of Complex Systems. Dordrecht: Springer Science & Business Media.

[B24] Grado L. L. Johnson M. D. Netoff T. I. (2018). Bayesian adaptive dual control of deep brain stimulation in a computational model of parkinson's disease. PLoS Comput. Biol. 14:e1006606. 10.1371/journal.pcbi.1006606 30521519 PMC6298687

[B25] Hahn P. McIntyre C. (2010). Modeling shifts in the rate and pattern of subthalamopallidal network activity during deep brain stimulation. J. Comput. Neurosci. 28, 425–441. 10.1007/s10827-010-0225-8 20309620 PMC2881193

[B26] Haidar I. Pasillas-Lépine W. Chaillet A. Panteley E. Palfi S. Senova S. (2016). Closed-loop firing rate regulation of two interacting excitatory and inhibitory neural populations of the basal ganglia. Biol. Cybern. 110, 55–71. 10.1007/s00422-015-0678-y 26837751

[B27] Hammond C. Bergman H. Brown P. (2007). Pathological synchronization in Parkinson's disease: networks, models and treatments. Trends Neurosci. 30, 357–364. 10.1016/j.tins.2007.05.004 17532060

[B28] Hines M. L. Carnevale N. T. (1997). The NEURON simulation environment. Neural Comput. 9, 1179–1209. 10.1162/neco.1997.9.6.1179 9248061

[B29] Ioannou P. Fidan B. (2006). Adaptive Control Tutorial. Advances in Design and Control. Philadelphia, PA: SIAM.

[B30] Ioannou P. Kokotovic P. V. (1984). Instability analysis and improvement of robustness of adaptive control. Automatica 20, 583–594. 10.1016/0005-1098(84)90009-8

[B31] Kang G. Lowery M. (2013). Interaction of oscillations, and their suppression via deep brain stimulation, in a model of the cortico-basal ganglia network. IEEE Trans. Neural Syst. Rehabil. Eng. 21, 244–253. 10.1109/TNSRE.2013.2241791 23476006

[B32] Kang G. Lowery M. (2014). Effects of antidromic and orthodromic activation of STN afferent axons during dbs in parkinson's disease: a simulation study. Front. Comput. Neurosci. 8:32. 10.3389/fncom.2014.00032 24678296 PMC3958751

[B33] Koelman L. A. Lowery M. M. (2019). Beta-band resonance and intrinsic oscillations in a biophysically detailed model of the subthalamic nucleus-globus pallidus network. Front. Comput. Neurosci. 13:77. 10.3389/fncom.2019.00077 31749692 PMC6848887

[B34] Kühn A. A. Kempf F. Brücke C. Doyle L. G. Martinez-Torres I. Pogosyan A.. (2008). High-frequency stimulation of the subthalamic nucleus suppresses oscillatory β activity in patients with Parkinson's disease in parallel with improvement in motor performance. J. Neurosci. 28, 6165–6173. 10.1523/JNEUROSCI.0282-08.2008 18550758 PMC6670522

[B35] Kühn A. A. Kupsch A. Schneider G. H. Brown P. (2006). Reduction in subthalamic 8-35 Hz oscillatory activity correlates with clinical improvement in Parkinson's disease. Eur. J. Neurosci. 23, 1956–1960. 10.1111/j.1460-9568.2006.04717.x 16623853

[B36] Kumaravelu K. Brocker D. Grill W. (2016). A biophysical model of the cortex-basal ganglia-thalamus network in the 6-ohda lesioned rat model of parkinson's disease. J. Comput. Neurosci. 40, 207–229. 10.1007/s10827-016-0593-9 26867734 PMC4975943

[B37] Li Q. Ke Y. Chan D. Qian Z. Yung K. Ko H.. (2012). Therapeutic deep brain stimulation in Parkinsonian rats directly influences motor cortex. Neuron 76, 1030–1041. 10.1016/j.neuron.2012.09.032 23217750

[B38] Little S. Beudel M. Zrinzo L. Foltynie T. Limousin P. Hariz M.. (2016). Bilateral adaptive deep brain stimulation is effective in Parkinson's disease. J. Neurol. Neurosurg. Psychiatry 87, 717–721. 10.1136/jnnp-2015-310972 26424898 PMC4941128

[B39] Little S. Pogosyan A. Neal S. Zavala B. Zrinzo L. Hariz M.. (2013). Adaptive deep brain stimulation in advanced Parkinson disease. Ann. Neurol. 74, 449–457. 10.1002/ana.23951 23852650 PMC3886292

[B40] Litvak V. Jha A. Eusebio A. Oostenveld R. Foltynie T. Limousin P.. (2011). Resting oscillatory cortico-subthalamic connectivity in patients with Parkinson's disease. Brain 134, 359–374. 10.1093/brain/awq332 21147836

[B41] Liu C. Zhou C. Wang J. Fietkiewicz C. Loparo K. A. (2020). The role of coupling connections in a model of the cortico-basal ganglia-thalamocortical neural loop for the generation of beta oscillations. Neural Netw. 123, 381–392. 10.1016/j.neunet.2019.12.021 31911186

[B42] Liu C. Zhu Y. Liu F. Wang J. Li H. Deng B.. (2017). Neural mass models describing possible origin of the excessive beta oscillations correlated with parkinsonian state. Neural Netw. 88, 65–73. 10.1016/j.neunet.2017.01.011 28192762

[B43] Lozano A. M. Lipsman N. Bergman H. Brown P. Chabardes S. Chang J. W.. (2019). Deep brain stimulation: current challenges and future directions. Nat. Rev. Neurol. 15, 148–160. 10.1038/s41582-018-0128-2 30683913 PMC6397644

[B44] Magill P. J. Bolam J. P. Bevan M. D. (2001). Dopamine regulates the impact of the cerebral cortex on the subthalamic nucleus-globus pallidus network. Neuroscience 106, 313–330. 10.1016/S0306-4522(01)00281-0 11566503

[B45] Mallet N. Pogosyan A. Márton L. F. Bolam J. P. Brown P. Magill P. J. (2008). Parkinsonian beta oscillations in the external globus pallidus and their relationship with subthalamic nucleus activity. J. Neurosci. 52, 14245–14258. 10.1523/JNEUROSCI.4199-08.2008 19109506 PMC4243385

[B46] McConnell G. So R. Hilliard J. Lopomo P. Grill W. M. (2012). Effective deep brain stimulation suppresses low-frequency network oscillations in the basal ganglia by regularizing neural firing patterns. J. Neurosci. 32, 15657–15668. 10.1523/JNEUROSCI.2824-12.2012 23136407 PMC3502634

[B47] McManus L. Flood M. Lowery M. (2019). Beta-band motor unit coherence and nonlinear surface emg features of the first dorsal interosseous muscle vary with force. J. Neurophysiol. 122, 1147–1162. 10.1152/jn.00228.2019 31365308 PMC6766730

[B48] Nevado-Holgado A. J. Mallet N. Magill P. J. Bogacz R. (2014). Effective connectivity of the subthalamic nucleus-globus pallidus network during parkinsonian oscillations. J. Physiol. 592, 1429–1455. 10.1113/jphysiol.2013.259721 24344162 PMC3979604

[B49] Nevado-Holgado A. J. Terry J. R. Bogacz R. (2010). Conditions for the generation of beta oscillations in the subthalamic nucleus-globus pallidus network. J. Neurosci. 30, 12340–12352. 10.1523/JNEUROSCI.0817-10.2010 20844130 PMC6633459

[B50] Obeso J. Olanow C. Rodriguez-Oroz M. Krack P. Kumar R. Lang A. (2001). Deep-brain stimulation of the subthalamic nucleus or the pars interna of the globus pallidus in Parkinson's disease. New Engl. J. Med. 345, 956–963. 10.1056/NEJMoa000827 11575287

[B51] Orłowski J. (2019). Adaptive control of time-delay systems to counteract pathological brain oscillations (Ph.D. thesis). Université Paris-Saclay, Saint-Aubin.

[B52] Otsuka T. Abe T. Song W. (2004). Conductance-based model of the voltage-dependent generation of a plateau potential in subthalamic neurons. J. Neurophysiol. 92, 255–264. 10.1152/jn.00508.2003 15212440

[B53] Parastarfeizabadi M. Kouzani A. Z. (2017). Advances in closed-loop deep brain stimulation devices. J. Neuroeng. Rehabil. 14:79. 10.1186/s12984-017-0295-1 28800738 PMC5553781

[B54] Pasillas-Lépine W. (2013). Delay-induced oscillations in Wilson and Cowan's model: an analysis of the subthalamo-pallidal feedback loop in healthy and parkinsonian subjects. Biol. Cybern. 107, 289–308. 10.1007/s00422-013-0549-3 23400597

[B55] Pasillas-Lépine W. Haidar I. Chaillet A. Panteley E. (2013). “Closed-loop deep brain stimulation based on firing-rate regulation,” in 6th International IEEE/EMBS Conference on Neural Engineering (NER) (San Diego, CA), 166–169.

[B56] Pavlides A. John Hogan S. Bogacz R. (2012). Improved conditions for the generation of beta oscillations in the subthalamic nucleus-globus pallidus network. Eur. J. Neurosci. 36, 2229–2239. 10.1111/j.1460-9568.2012.08105.x 22805067

[B57] Pavlov A. van de Wouw N. Nijmeijer H. (2007). Frequency response functions for nonlinear convergent systems. IEEE Trans. Autom. Control 52, 1159–1165. 10.1109/TAC.2007.899020

[B58] Plenz D. Kital S. T. (1999). A basal ganglia pacemaker formed by the subthalamic nucleus and external globus pallidus. Nature 400, 677–682. 10.1038/23281 10458164

[B59] Popovych O. V. Tass P. A. (2019). Adaptive delivery of continuous and delayed feedback deep brain stimulation - a computational study. Sci. Rep. 9, 1–17. 10.1038/s41598-019-47036-4 31332226 PMC6646395

[B60] Pospischil M. Toledo-Rodriguez M. Monier C. Piwkowska Z. Bal T. Frégnac Y.. (2008). Minimal hodgkin–huxley type models for different classes of cortical and thalamic neurons. Biol. Cybern. 99, 427–441. 10.1007/s00422-008-0263-8 19011929

[B61] Rosa M. Arlotti M. Ardolino G. Cogiamanian F. Marceglia S. Di Fonzo A.. (2015). Adaptive deep brain stimulation in a freely moving parkinsonian patient. Mov. Disord. 30, 1003–1005. 10.1002/mds.26241 25999288 PMC5032989

[B62] Rosin B. Slovik M. Mitelman R. Rivlin-Etzion M. Haber S. N. Israel Z.. (2011). Closed-loop deep brain stimulation is superior in ameliorating parkinsonism. Neuron 72, 370–384. 10.1016/j.neuron.2011.08.023 22017994

[B63] Rossi L. Foffani G. Marceglia S. Bracchi F. Barbieri S. Priori A. (2007). An electronic device for artefact suppression in human local field potential recordings during deep brain stimulation. J. Neural Eng. 4, 96–106. 10.1088/1741-2560/4/2/010 17409484

[B64] Rubin J. Terman D. (2004). High frequency stimulation of the subthalamic nucleus eliminates pathological thalamic rhythmicity in a computational model. J. Comput. Neurosci. 16, 211–235. 10.1023/B:JCNS.0000025686.47117.67 15114047

[B65] Santaniello S. Fiengo G. Glielmo L. Grill W. M. (2010). Closed-loop control of deep brain stimulation: a simulation study. IEEE Trans. Neural Syst. Rehabil. Eng. 19, 15–24. 10.1109/TNSRE.2010.2081377 20889437

[B66] Santos F. J. Costa R. M. Tecuapetla F. (2011). Stimulation on demand: closing the loop on deep brain stimulation. Neuron 72, 197–198. 10.1016/j.neuron.2011.10.004 22017983

[B67] Shah S. A. Tinkhauser G. Chen C. C. Little S. Brown P. (2018). “Parkinsonian tremor detection from subthalamic nucleus local field potentials for closed-loop deep brain stimulation,” in Proceedings of the Annual International Conference of the IEEE Engineering in Medicine and Biology Society, EMBS (Honolulu, HI), 2320–2324. 10.1109/EMBC.2018.8512741 PMC6277049 30440871

[B68] Sharott A. Magill P. J. Harnack D. Kupsch A. Meissner W. Brown P. (2005). Dopamine depletion increases the power and coherence of β-oscillations in the cerebral cortex and subthalamic nucleus of the awake rat. Eur. J. Neurosci. 21, 1413–1422. 10.1111/j.1460-9568.2005.03973.x 15813951

[B69] Shouno O. Tachibana Y. Nambu A. Doya K. (2017). Computational model of recurrent subthalamo-pallidal circuit for generation of parkinsonian oscillations. Front. Neuroanat. 11:21. 10.3389/fnana.2017.00021 28377699 PMC5359256

[B70] Stanslaski S. Afshar P. Cong P. Giftakis J. Stypulkowski P. Carlson D.. (2012). Design and validation of a fully implantable, chronic, closed-loop neuromodulation device with concurrent sensing and stimulation. IEEE Trans. Neural Syst. Rehabil. Eng. 20, 410–421. 10.1109/TNSRE.2012.2183617 22275720

[B71] Su F. Kumaravelu K. Wang J. Grill W. M. (2019). Model-based evaluation of closed-loop deep brain stimulation controller to adapt to dynamic changes in reference signal. Front. Neurosci. 13:956. 10.3389/fnins.2019.00956 31551704 PMC6746932

[B72] Tachibana Y. Iwamuro H. Kita H. Takada M. Nambu A. (2011). Subthalamo-pallidal interactions underlying parkinsonian neuronal oscillations in the primate basal ganglia. Eur. J. Neurosci. 34, 1470–1484. 10.1111/j.1460-9568.2011.07865.x 22034978

[B73] Tachibana Y. Kita H. Chiken S. Takada M. Nambu A. (2008). Motor cortical control of internal pallidal activity through glutamatergic and gabaergic inputs in awake monkeys. Eur. J. Neurosci. 27, 238–253. 10.1111/j.1460-9568.2007.05990.x 18093168

[B74] Terman D. Rubin J. Yew A. Wilson C. (2002). Activity patterns in a model for the subthalamopallidal network of the basal ganglia. J. Neurosci. 22, 2963–2976. 10.1523/JNEUROSCI.22-07-02963.2002 11923461 PMC6758326

[B75] Tukhlina N. Rosenblum M. Pikovsky A. Kurths J. (2007). Feedback suppression of neural synchrony by vanishing stimulation. Phys. Rev. E 75:11918. 10.1103/PhysRevE.75.011918 17358195

[B76] Velisar A. Syrkin-Nikolau J. Blumenfeld Z. Trager M. Afzal M. Prabhakar V.. (2019). Dual threshold neural closed loop deep brain stimulation in Parkinson disease patients. Brain Stimul. 12, 868–876. 10.1016/j.brs.2019.02.020 30833216

[B77] Volkmann J. Herzog J. Kopper F. Deuschl G. (2002). Introduction to the programming of deep brain stimulators. Mov. Disord. 17, S181–S187. 10.1002/mds.10162 11948775

[B78] West T. O. Berthouze L. Halliday D. M. Litvak V. Sharott A. Magill P. J.. (2018). Propagation of beta/gamma rhythms in the cortico-basal ganglia circuits of the parkinsonian rat. J. Neurophysiol. 119, 1608–1628. 10.1152/jn.00629.2017 29357448 PMC6008089

[B79] Yeganefar N. Pepe P. Dambrine M. (2008). Input-to-State Stability of time-delay systems: a link with exponential stability. IEEE Trans. Autom. Control 53, 1526–1531. 10.1109/TAC.2008.928340

