# Supplementary material for: Self-Tuning Deep Brain Stimulation Controller for Suppression of Beta Oscillations: Analytical Derivation and Numerical Validation
Source: Front Neurosci. 2020 Jun 30;14:639. doi: 10.3389/fnins.2020.00639 (PMC7339866; doi:10.3389/fnins.2020.00639)
Supplement: Supplementary file 1 [file Data_Sheet_1.PDF]

Supplementary Material

1 Supplementary Figures

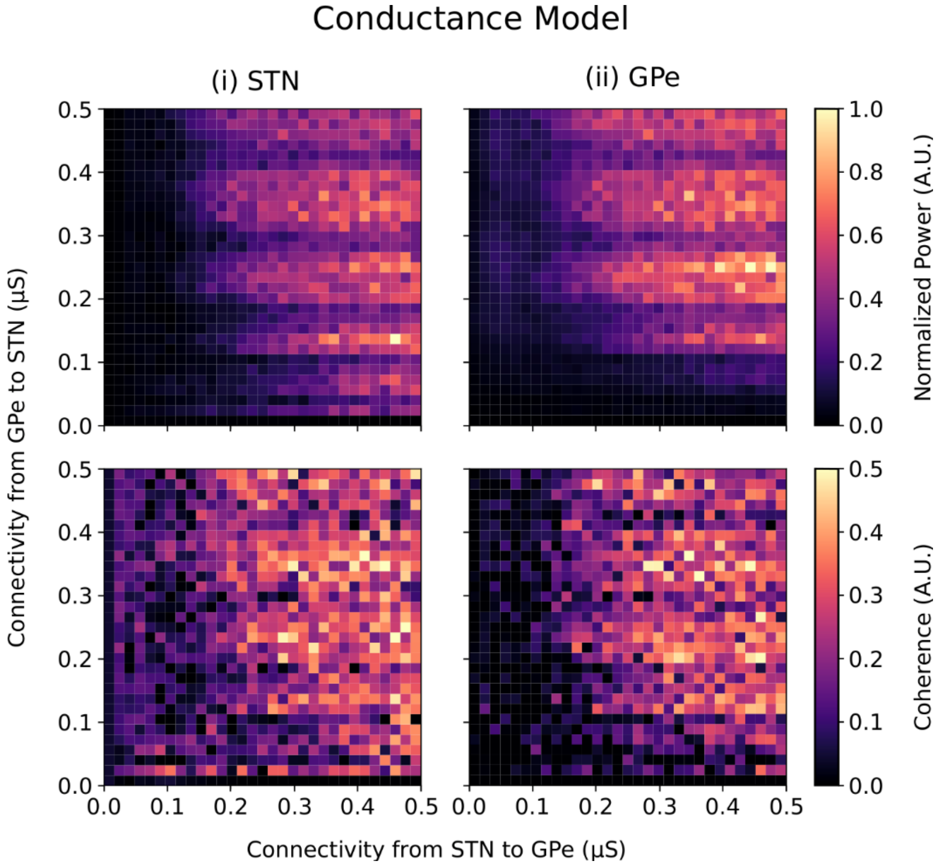

Supplementary Figure 1. Normalized endogenous beta-band activity in the conductance-based model for varying STN and GPe coupling strengths. Beta-band activity was quantified as the power and coherence of the cumulative spike trains for each population located between 16 - 24 Hz. Beta-band power in each population was estimated by integrating the power spectra of the cumulative spike trains for the population between 16 - 24 Hz. The beta-band power for each population was normalized between 0 and 1 in each panel separately. The beta-band coherence of each population was estimated from pairs of composite spike trains randomly selected from the respective population. The selected spike trains were divided into two groups with an equal number of trains. The spike trains in each group were summed to obtain two composite spike trains. The magnitude squared coherence between the two composite spike trains was then calculated with 1 s overlapping Hamming windows. This was repeated for 200 randomly chosen combinations of the selected population spike trains, as each combination will generate a slightly different coherence estimate. The coherence estimate for the population at each pair of coupling strength values was subsequently the median coherence spectrum over all 200 combinations at the respective coupling strength value. The beta-band coherence was determined by integrating the resulting coherence spectrum between 16 – 24 Hz.

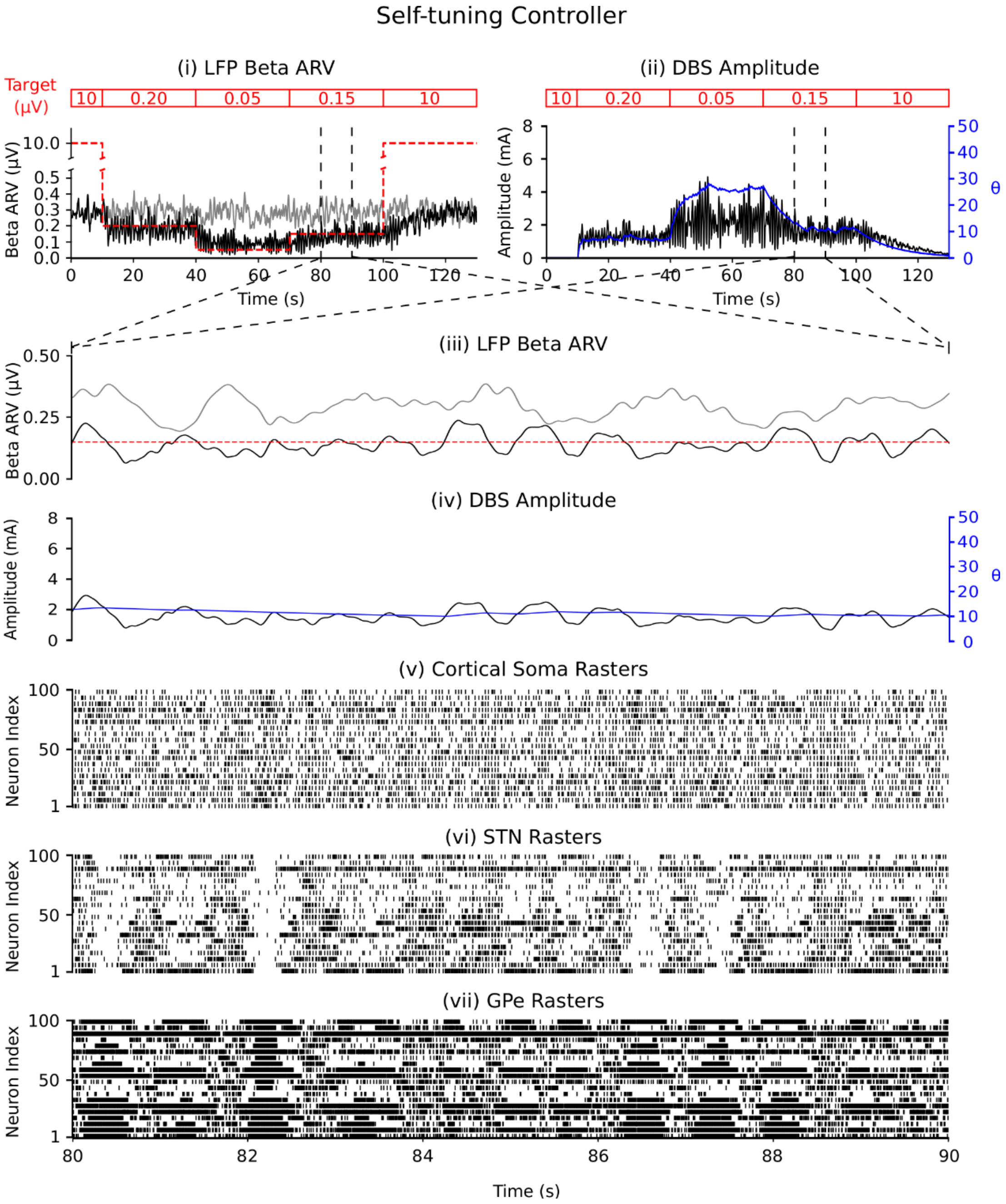

Supplementary Figure 2. Conductance-based model Raster Plots. (i) and (ii) correspond to panels A(i) and (ii) in Figure 6 of the main manuscript. The behavior of the conductance-based model during the 80 – 90 s simulation segment are presented in (iii) – (vii). (iii) The LFP beta ARV. The grey line represents when DBS is off, the black line represents when the self-tuning algorithm is on during this period, with the target value being illustrated as the red dotted line. (iv) DBS amplitude and controller gain. The black line corresponds to the resulting DBS amplitude during this period, where the self-tuning controller is implemented. The gain of the self-tuning controller for this period is represented by the blue line. (v-vii) Population raster plots. These panels represent the activity of the cortical soma, STN and GPe populations during the 80 – 90 s simulation period. For illustration, every fifth neuron from the populations are illustrated. During DBS cortical somas desynchronize, STN neurons are suppressed and GPe neurons entrain to the stimulation.
